# Pediatric Intensive Care Nurse Staffing Measures and Patient Outcomes During the COVID-19 Pandemic

**DOI:** 10.1001/jamanetworkopen.2025.15376

**Published:** 2025-06-12

**Authors:** Warren Michael Taylor, Jonathan Pelletier, Julia A. Heneghan, Sriram Ramgopal, Kelly M. Toth, Andrew Prout, Maeve Woeltje, Gabriella Butler, Anthony Fabio, Idris Evans, Richard Brilli, Patrick M. Kochanek, Robert S. B. Clark, Christopher M. Horvat

**Affiliations:** 1 UPMC Children’s Hospital of Pittsburgh, Pittsburgh, Pennsylvania; 2 Department of Critical Care Medicine, University of Pittsburgh School of Medicine, Pittsburgh, Pennsylvania; 3 Safar Center for Resuscitation Research, University of Pittsburgh, Pittsburgh, Pennsylvania; 4 Division of Pediatric Critical Care Medicine, Department of Pediatrics, Akron Children’s Hospital, Akron, Ohio; 5 Division of Pediatric Critical Care Medicine, Department of Pediatrics, University of Minnesota, Minneapolis; 6 Division of Emergency Medicine, Department of Pediatrics, Ann & Robert H. Lurie Children’s Hospital of Chicago, Chicago, Illinois; 7 Division of Pediatric Critical Care Medicine, Department of Pediatrics, Children’s Hospital of Michigan, Detroit; 8 Epidemiology Data Center, University of Pittsburgh School of Public Health, Pittsburgh, Pennsylvania; 9 Division of Pediatric Critical Care Medicine, Department of Pediatrics, Nationwide Children’s Hospital, Columbus, Ohio

## Abstract

**Importance:**

During the COVID-19 pandemic, pediatric intensive care units (PICUs) reportedly relied more on temporary contract nurses hired through staffing agencies (agency nurses) to fill gaps related to increased nurse turnover. It is unknown whether these changes in nurse staffing were associated with patient outcomes.

**Objective:**

To determine whether the proportion of PICU agency staff or nurse turnover was associated with patient outcomes during the pandemic.

**Design, Setting, and Participants:**

This retrospective, multicenter, linked database cross-sectional study was performed at PICUs in the US between January 1, 2019, and December 31, 2022. Participants included patients younger than 19 years admitted to a PICU with data in both the Children’s Hospital Association Pediatric Health Information Systems and PROSPECT Hospital Essentials databases. Data were analyzed from March 15, 2023, to August 1, 2024.

**Exposure:**

The yearly proportion of agency nurses and nurse turnover.

**Main Outcomes and Measures:**

Descriptive characteristics were examined for PICUs grouped by the median proportion of agency nurses or nursing turnover each year. A variable staffing metric was defined as greater than 10% change in the proportion of agency nurses from the intrahospital baseline in 2019. Multivariable negative binomial models controlling for illness severity and hospital effect with median yearly staffing metrics examined whether the proportion of agency nurses or nurse turnover was associated with mortality, PICU complications, central line–associated bloodstream infections, or ventilator-associated pneumonias.

**Results:**

There were 218 789 admissions across 20 PICUs, with a median patient age of 45 (IQR, 9-138) months (119 997 [54.8%] male). PICUs with a variable proportion of agency nurses had significantly more patients with PICU complications (9695 of 120 017 [8.1%] vs 7141 of 98 772 [7.2%]; *P* < .001). Multivariable analysis indicated an increase of 19 (95% CI, 7-39) ventilator-associated pneumonias per 10 000 ventilator-days in 2020. For a PICU with 10% higher nurse turnover than the median turnover, there were 10 (95% CI, 8-13) more PICU complications per 10 000 patient-days in 2022.

**Conclusions and Relevance:**

In this cross-sectional study of nurse staffing measures and patient outcomes during COVID-19, complex associations existed throughout the study years. More data are needed to determine optimal staffing strategies during times of workforce strain.

## Key Points

**Question:**

During the COVID-19 pandemic, were pediatric intensive care unit (PICU) nurse staffing measures associated with patient outcomes?

**Findings:**

In this cross-sectional linked database study including 218 789 PICU admissions, PICU nurse staffing measures were not associated with selected patient outcomes in 2019. PICUs with a variable proportion of agency nurses during the study period had significantly more PICU complications. The proportion of agency nurses on staff was associated with ventilator-associated pneumonia in 2020. Nurse turnover was associated with a composite of all PICU complications in 2022.

**Meaning:**

These findings suggest that during the COVID-19 pandemic, nurse staffing measures demonstrated a complex pattern of associations with PICU patient outcomes.

## Teaser

This cross-sectional study assesses whether agency nurse staffing and nurse turnover in pediatric intensive care units were associated with patient outcomes during the COVID-19 pandemic in the US.

## Introduction

The COVID-19 public health emergency was associated with higher rates of health care staff turnover and an increase in position vacancies.^1,2^ A survey by the American Organization for Nursing Leadership reported that staff recruitment, retention, and the emotional health and well-being of staff were the top concerns of nurse leaders during the COVID-19 pandemic.^3^ Many health systems turned to temporary contract nurses hired through staffing agencies, or agency nurses, to fulfill essential patient care duties.^4,5^ During the pandemic, agency nurse positions increased from approximately 10 000 in early 2020 to nearly 50 000 in September 2021.^6^

The use of agency nurses has been identified as a potential threat to patient safety by the National Academy of Medicine.^7,8^ Agency nurses are less familiar with an organization’s care policies, procedures, and personnel than staff nurses. This can be amplified by truncated orientation periods intended to quickly incorporate agency staff into front-line care to address acute staffing strains.^7^ Despite these concerns, data are mixed regarding whether the use of agency nurses affects patient outcomes. A recent meta-analysis^9^ noted a lack of a consistent relationship between agency nurses and patient outcomes, in part due to confounding related to risk factors such as varied staffing levels and characteristics of particular care environments. Higher patient to nurse ratios, for example, are associated with increased nurse burnout and job dissatisfaction,^10,11^ patient mortality,^12,13,14,15^ ventilator-associated pneumonia (VAP),^16,17^ and central line–associated bloodstream infection (CLABSI).^13,14,15^ Improved patient to nurse ratios are associated with decreased pressure ulcers, medication errors, restraint use, unplanned extubations, iatrogenic infections, and mortality.^16,17,18,19,20,21,22,23,24,25^

Prior research investigating the relationship between nurse staffing and patient outcomes^19,20,21,22,23,24,25^ has largely been performed in nonpediatric environments. During the pandemic, children’s hospitals also experienced staffing strains related to increased turnover of front-line nurses in combination with atypical cycles of common childhood illnesses such as respiratory syncytial virus.^26,27^ Overall lower patient volumes, rates of complications, and mortality in children’s hospitals compared with adult care settings pose a challenge to identifying potentially rare but meaningful associations between nurse staffing measures and outcomes among acutely ill children. Whether the use of agency nurses or the related staffing strains that led to relying on agency nurses were associated with patient outcomes in the pediatric intensive care unit (PICU) during the pandemic is unknown. Therefore, we sought to determine whether PICU agency nurse staffing or PICU nurse turnover was associated with patient outcomes during the COVID-19 pandemic in the US using 2 multicenter databases. We hypothesized that a greater proportion of agency nurses and greater nursing turnover would each be associated with more patient care complications and higher mortality.

## Methods

### Study Design and Data Sources

We performed a retrospective, cross-sectional study of admissions to PICUs at US children’s hospitals between January 1, 2019, and December 31, 2022. Data were collated using 2 datasets from the Children’s Hospital Association (CHA): the Pediatric Health Information Systems (PHIS) and PROSPECT Hospital Essentials (PROSPECT) databases. PHIS harbors patient-level data while PROSPECT harbors hospital- and unit-level human resources measures. PHIS hospital encounters during the study years were selected if the patient was admitted to the PICU during the admission. All hospitals studied provided yearly survey data to the PROSPECT database for the study duration. Among study hospitals with multiple PICUs, the main PICU within each institution’s main campus, as designated in PROSPECT, was examined in this study and matched to the PHIS patients. Patient-level PHIS data were linked to PICU-level PROSPECT data using hospital number. The University of Pittsburgh Institutional Review Board deemed the study to be exempt from approval and informed consent based on the use of deidentified patient and hospital data. This study was completed in accordance with the Strengthening the Reporting of Observational Studies in Epidemiology (STROBE) guidelines.

### Data Acquisition

We included all encounters for patients younger than 19 years admitted to PICUs, excluding patients in stand-alone cardiac ICUs during the study period. Demographic data included hospital-reported patient age, admission date, sex, race, and complex chronic condition flags. Race was categorized as American Indian or Alaska Native, Asian, Black, Native Hawaiian or Other Pacific Islander, White, or other. “Other” included races reported by PHIS-member hospitals that were not one of the predefined PHIS race categories. Race is reported in this study to describe the diversity of the study population as one indication of the potential generalizable nature of the findings. Precalculated variables included severity level (based off the entire admission using the All Patient Refined Diagnosis Related Groups severity of illness score^28^) and expected length of stay (LOS; a CHA-derived calculation).^29^ Encounter data from the entire hospitalization were included. The main outcomes of interest were mortality and complications of PICU care. Medical and surgical complications are separate data elements in PHIS that are indicators of whether an *International Statistical Classification of Disease, Tenth Revision* (*ICD-10*), code associated with a care-related complication, such as VAP, is present for an encounter. We created an author-defined indicator of PICU complications by further curating the CHA medical and surgical complication codes (eTable 1 in Supplement 1). In addition, CLABSI and VAP were individually evaluated as outcomes. Additional outcomes of interest included extracorporeal membrane oxygenation (ECMO), any renal replacement therapy (RRT), hospital LOS, LOS ratio (calculated as actual divided by expected LOS), PICU LOS, vasoactive medication use, and in-hospital cardiac arrest during admission (using *ICD-10* codes).

Staffing measures included yearly data on registered nurse turnover, defined as the number of PICU employees with the job title nurse who were terminated or left voluntarily during the reporting period divided by the total number of active nurses, and the proportion of agency nurses. Agency staffing was defined as the proportion of agency nurses per hospital PICU and does not represent the part-time nursing staff primarily employed by the respective PICU in the PROSPECT database.

PICUs were classified into 4 groups based on the proportion of agency nurses: group 1 (0), group 2 (0.1%-5.0%), group 3 (5.1%-10.0%), and group 4 (>10.0%). Hospitals were also grouped by annual nurse turnover: group 1 (<12.5%), group 2 (12.5%-14.9%), group 3 (15.0%-17.4%), and group 4 (≥17.5%) (eMethods in Supplement 1). To evaluate staffing trends during the study period, we used alluvial diagrams for agency nurse proportions and nurse turnover. Given variability in reported turnover rates and market differences, nurse turnover data were normalized to the median across all hospitals. Multivariable modeling used actual event rates and nursing metrics for each hospital, with 2019 prepandemic agency staffing as the baseline for agency models. Since some hospitals had higher baseline agency nurse proportions and greater experience integrating them, we examined whether variability in agency staffing influenced outcomes. Hospitals were categorized as stable (<10% change) or variable (≥10% change) in agency nurse proportion from their 2019 baseline. Variability thresholds were set using the 2019 mean agency proportion and SD across hospitals (eMethods in Supplement 1).

### Statistical Analysis

Data were analyzed from March 15, 2023, to August 1, 2024. We analyzed nonnormally distributed continuous data using the Kruskal-Wallis rank sum test to assess for overall differences between groups and presented these as medians and IQRs. Continuous variables with normal distributions were presented as means with SDs. We evaluated dichotomous variables with Pearson χ^2^ testing similarly to assess for overall differences between groups, with findings expressed as percentages. We developed separate models to analyze mortality and complications. Given the count nature, heteroskedasticity, excessive variance, and overdispersion of the data, outcomes were analyzed with negative-binomial regression models.^30^ A log-offset for patient-days was used for models examining PICU complications as the outcome, a log-offset for number of admissions for models examining mortality as the outcome, and a log-offset for patient ventilator-days for models examining VAP as the outcome (eMethods in Supplement 1). Because staffing practices, disease patterns, patient volume, and societal stressors varied yearly, separate models were created for each year of the study period. Hospitals differed in reporting nurse turnover proportion throughout the study period; therefore, in years when hospitals did not report these data, they were excluded from that year’s nurse turnover analysis. Only PHIS records with complete data were included.

We created multivariable models that included patient severity of illness as a fixed effect and hospital as a random effect. The primary exposure variables, proportion of agency and nursing turnover, were modeled as continuous variables to maintain the full variability in the data and avoid data loss with arbitrary cutoffs. We presented results using incidence rate ratios (IRRs) based on negative binomial regression estimates with 95% CIs.^31^ To translate IRRs into a graphical depiction of event rates with varied levels of nursing metrics, significant multivariable models were then used to estimate hypothetical outcomes. The hypothetical unit was created using a fixed yearly mean illness severity, patient hospital days, ventilator-days, or admissions for all units, as appropriate, and events were estimated based on varying proportion of agency and nurse turnover in each model. Nurse turnover model estimations were evaluated by comparing the hypothetical unit’s percentage of nurse turnover change to the yearly median across all units.

To adjust for multiple comparisons, statistical significance was defined using a Bonferroni-corrected threshold of 2-sided *P* < .006. All analyses were performed in R, version 4.3.3 (R Foundation for Statistical Computing). Code drafting and debugging were performed with assistance from artificial intelligence (ChatGPT, versions 3.5 and 4o mini; OpenAI).

## Results

### Agency Proportions, Patient Characteristics, and Outcomes 

There were 218 789 admissions from 20 academic PICUs across the 4-year study period. Table 1 shows patient characteristics for the 4 groups of agency staffing. The median age of included patients was 45 (IQR, 9-138) months; 98 664 (45.1%) were female, 119 997 (54.8%) were male, and 128 (0.1%) were unspecified sex. In terms of race, 9170 (4%) were Asian; 1092 (0.5%), American Indian or Alaska Native; 42 313 (19.3%), Black; 1268 (0.6%), Native Hawaiian or Other Pacific Islander; 134 811 (61.6%), White; and 22 952 (10.5%), other race. A total of 140 500 admissions (64.2%) were for patients with at least 1 complex chronic condition. As demonstrated in Table 2, group 4 PICUs, which had the highest proportion of agency nurses, had the lowest mean severity of illness. The 59 838 admissions in group 4 also had the shortest LOS (median, 4 [IQR, 2-8] days), the lowest LOS ratio (median, 0.80 [IQR, 0.49-1.26]), fewer PICU days across the study years (median, 2 [IQR, 1-4] days), and fewer cases requiring RRT (565 [0.9%]) or ECMO (598 [1.0%]), experiencing cardiac arrests (357 [0.6%]), and receiving vasoactive infusions (10 825 [18.1%]) compared with the other groups. A total of 16 836 patients (7.7%) experienced 21 033 PICU complications. Group 1, which did not use any agency staffing during the study period, had the lowest proportion of patients who experienced PICU complications (1355 of 19 932 [6.8%]). Overall, patient characteristics and outcomes across groups defined by the proportion of agency nurses for each year of the study period are displayed in eTables 2 to 9 in Supplement 1. Median nurse turnover varied significantly across the agency nurse groups for all study years and was greater later in the COVID-19 public health emergency, at 13.0% (IQR, 10.5%-14.9%) in 2019, 12.4% (IQR, 11.4%-14.3%) in 2020, 18.5% (IQR, 16.8%-22.9%) in 2021, and 17.6% (IQR, 15.0%-19.7%) in 2022.

**Table 1.** Patient Demographic Characteristics

Characteristic	Admission group^a^				
	Overall	Group 1 (0%)	Group 2 (0.1%-5.0%)	Group 3 (5.1%-10.0%)	Group 4 (>10.0%)
No. of admissions	218 789	19 932	99 036	39 983	59 838
No. of PICUs	20	6	6	5	3
Age at admission, median (IQR), mo	45 (9-138)	43 (9-133)	41 (8-134)	49 (11-141)	52 (12-144)
Sex, No. (%)					
Female	98 664 (45.1)	8958 (44.9)	44 654 (45.1)	17 898 (44.8)	27 154 (45.4)
Male	119 997 (54.8)	10 972 (55.0)	54 340 (54.9)	22 026 (55.1)	32 659 (54.6)
Unspecified	128 (<0.1)	2 (<0.1)	42 (<0.1)	59 (0.1)	25 (<0.1)
Complex chronic condition, No. (%)	140 500 (64.2)	11 434 (57.4)	65 607 (66.2)	25 990 (65.0)	37 469 (62.6)
Race, No. (%)^b^					
American Indian or Alaska Native	1092 (0.5)	95 (0.5)	292 (0.3)	203 (0.5)	502 (0.8)
Asian	9170 (4.2)	465 (2.3)	3407 (3.4)	1812 (4.5)	3486 (5.8)
Black	42 313 (19.3)	3909 (19.6)	24 134 (24.4)	9811 (24.5)	4459 (7.5)
Native Hawaiian or Other Pacific Islander	1268 (0.6)	59 (0.3)	218 (0.2)	228 (0.6)	763 (1.3)
White	134 811 (61.6)	14 352 (72.0)	57 438 (58.0)	24 666 (61.7)	38 355 (64.1)
Other	22 952 (10.5)	573 (2.9)	6637 (6.7)	3133 (7.8)	12 609 (21.1)
Nurse turnover, median (IQR), %	15.0 (12.4-18.1)	14.9 (13.9-15.0)	14.9 (11.5-17.6)	15.3 (12.1-17.8)	17.6 (13.4-20.9)
Severity level, mean (SD)^c^	2.91 (0.97)	2.82 (0.97)	2.95 (0.95)	3.07 (0.94)	2.77 (0.99)
Expected LOS, median (IQR), d	5 (3-11)	9 (3-9)	6 (3-12)	6 (3-12)	4 (3-9)

Abbreviations: LOS, length of stay; PICU, pediatric intensive care unit.

^a^ Groups are determined by the proportion of agency nurse staff given in parentheses. Data are included from 2019 to 2022.

^b^ Data were missing for 7183 patients; percentages reflect only patients with known data.

^c^ Defined using the All Patient Refined Diagnosis Related Groups classification by Pediatric Health Information Systems. It is determined based on secondary diagnoses, reflecting both comorbid conditions and the severity of the underlying illness, with scores ranging from 1 (minor) to 4 (extreme); higher scores indicate more severe illness and greater resource utilization.

**Table 2.** Outcomes Among All Patients and by Groups Defined by the Proportion of Agency Nurse Staff

Characteristic	Admission group^a^					*P* value
	Overall	Group 1 (0%)	Group 2 (0.1%-5.0%)	Group 3 (5.1%-10.0%)	Group 4 (>10.0%)	
No. of admissions	218 789	19 932	99 036	39 983	59 838	NA
LOS, median (IQR), d	4 (2-10)	4 (3-9)	5 (3-11)	5 (2-10)	4 (2-8)	<.001^b^
Length of stay ratio, median (IQR)	0.87 (0.52-1.37)	0.93 (0.59-1.47)	0.89 (0.56-1.40)	0.82 (0.49-1.36)	0.80 (0.49-1.26)	<.001^b^
PICU days, median (IQR)	2 (1-5)	2 (1-4)	2 (1-5)	2 (1-4)	2 (1-4)	<.001^b^
Patients with PICU complication, No. (%)	16 836 (7.7)	1129 (5.7)	8047 (8.1)	2280 (5.7)	4392 (7.3)	<.001^c^
No. of PICU complications	21 033	1335	10 281	3997	5420	NA
PICU complications, No. (%)						
CLABSIs	1839 (0.8)	181 (0.9)	783 (0.8)	339 (0.8)	536 (0.9)	.10^c^
VAPs	796 (0.4)	24 (0.1)	272 (0.3)	233 (0.6)	267 (0.4)	<.001^c^
Cardiac arrest	1561 (0.7)	139 (0.7)	728 (0.7)	337 (0.8)	357 (0.6)	<.001^c^
Renal replacement therapy	3143 (1.4)	268 (1.3)	1419 (1.4)	891 (2.2)	565 (0.9)	<.001^c^
Vasoactive infusion	55 481 (25.4)	6100 (30.6)	26 994 (27.3)	11 562 (28.9)	10 825 (18.1)	<.001^c^
ECMO	2279 (1.0)	207 (1.0)	1000 (1.0)	474 (1.2)	598 (1.0)	.02^c^
Mortality	5131 (2.3)	489 (2.5)	2447 (2.5)	1065 (2.7)	1130 (1.9)	<.001^c^

Abbreviations: CLABSI, central line–associated bloodstream infection; ECMO, extracorporeal membrane oxygenation; LOS, length of stay; NA, not applicable; PICU, pediatric intensive care unit; VAP, ventilator-associated pneumonia.

^a^ Groups are determined by the proportion of agency nurse staff given in parentheses. Data are included from 2019 to 2022.

^b^ Calculated using the Kruskal-Wallis rank sum test.

^c^ Calculated using the Pearson χ^2^ test.

As shown in Table 3, multivariable modeling demonstrated the proportion of agency nurse staff was associated with VAPs in 2020 (IRR, 1.13; 95% CI, 1.06-1.20; *P* < .001), CLABSIs in 2021 (IRR, 1.04; 95% CI, 1.01-1.07; *P* = .01), and all PICU complications in 2021 (IRR, 1.03; 95% CI, 1.01-1.06; *P* = .02), after adjusting for patient illness severity and controlling for center (hospital) effect. The adjusted models examining associations between the proportion of agency staff and outcomes indicate 19 (95% CI, 7-39) more VAPs per 10 000 patient-days from their baseline of 7 (95% CI, 5-9) VAPs per 10 000 patient-days in 2020 for a PICU with 10% agency nurse staff compared with a PICU with no agency nurse staff.

**Table 3.** Adjusted IRRs for the Proportion of Agency Nurses and Patient Outcomes^a^

Year	No. of PICUs	IRR (95% CI)			
		Mortality	PICU complications	CLABSIs	VAPs
2019	20	0.98 (0.95-1.01)	1.03 (0.99-1.05)	1.01 (0.97-1.05)	1.02 (0.94-1.12)
2020	20	0.98 (0.93-1.03)	1.04 (0.99-1.08)	1.03 (0.99-1.07)	1.13 (1.06-1.20)^b^
2021	19	0.99 (0.96-1.01)	1.03 (1.01-1.06)^c^	1.04 (1.01-1.07)^c^	1.05 (0.96-1.15)
2022	17	1.00 (0.98-1.01)	1.01 (0.99-1.02)	1.01 (0.99-1.03)	1.01 (0.98-1.05)

Abbreviations: CLABSI, central line–associated bloodstream infection; IRR, incidence rate ratio; PICU, pediatric intensive care unit; VAP, ventilator-associated pneumonia.

^a^ All models included a term for illness severity and hospital as a random effect. The mortality model included a log-offset term for patient admissions; the CLABSIs and PICU complications models, a log-offset term for patient hospital days; and the VAPs model, a log-offset term for ventilator-days.

^b^ Statistical significance as indicated by *P* < .006 determined by Bonferroni correction for the number of multivariable models.

^c^ Nominal significance as indicated by *P* < .05.

### Nurse Turnover, Patient Characteristics, and Outcomes 

Our analysis of yearly nurse turnover included 203 161 encounters from 20 academic PICUs that reported these data during the study period (eTables 10-19 in Supplement 1). A total of 15 613 patients experienced 19 496 PICU complications, and the proportion of patients experiencing complications differed significantly across nurse turnover groups (eTable 11 in Supplement 1). Group 3 (15.0%-17.5% nurse turnover) had the shortest LOS (median, 4 [IQR, 2-8] days), the lowest actual to estimated LOS ratio (median, 0.83 [IQR, 0.50-1.29]), the fewest PICU days (median, 2 [IQR, 1-4] days), the fewest patients who experienced PICU complications (4978 of 64 874 [7.7%]) or cardiac arrests (367 of 64 874 [0.6%]), and used less RRT (713 of 64 874 [1.1%]), vasoactive infusions (9829 of 64 874 [15.2%]), and ECMO (519 of 64 874 [0.8%]) (eTable 11 in Supplement 1).

In multivariable analysis (Table 4), nurse turnover was associated with PICU complications in 2022 (IRR, 1.03; 95% CI, 1.01-1.05; *P* = .001). The adjusted models examining associations between nurse turnover and outcomes found 10 (95% CI, 8-13) more PICU complications per 10 000 patient-days vs the baseline rate of 185 PICU complications per 10 000 patient-days in 2022 for a PICU with 10% greater than median turnover. The Figure displays estimated VAPs for a varied proportion of agency nurses in 2020 and PICU complications for nurse turnover in 2022 in a hypothetical PICU with mean illness severity and patient hospital-days.

**Table 4.** Adjusted IRRs for Nurse Turnover and Patient Outcomes^a^

Year	No. of PICUs	IRR (95% CI)			
		Mortality	PICU complications	CLABSIs	VAPs
2019	16	1.02 (0.96-1.07)	0.98 (0.96-1.01)	0.98 (0.92-1.05)	1.07 (0.92-1.25)
2020	17	1.07 (1.01-1.14)^b^	0.99 (0.94-1.04)	0.99 (0.93-1.05)	0.98 (0.86-1.12)
2021	18	1.01 (0.98-1.03)	1.01 (0.99-1.03)	1.00 (0.96-1.04)	0.96 (0.86-1.07)
2022	16	1.02 (0.98-1.07)	1.03 (1.01-1.05)^c^	0.99 (0.94-1.04)	1.04 (0.93-1.15)

Abbreviations: CLABSI, central line–associated bloodstream infection; IRR, incidence rate ratio; PICU, pediatric intensive care unit; VAP, ventilator-associated pneumonia.

^a^ All models included a term for illness severity and hospital as a random effect. The mortality model included a log-offset term for patient admissions; the CLABSIs and PICU complications models, a log-offset term for patient hospital days; and the VAPs model, a log-offset term for ventilator-days.

^b^ Nominal significance as indicated by *P* < .05.

^c^ Statistical significance as indicated by *P* < .006 determined by Bonferroni correction for the number of multivariable models.

**Figure.**
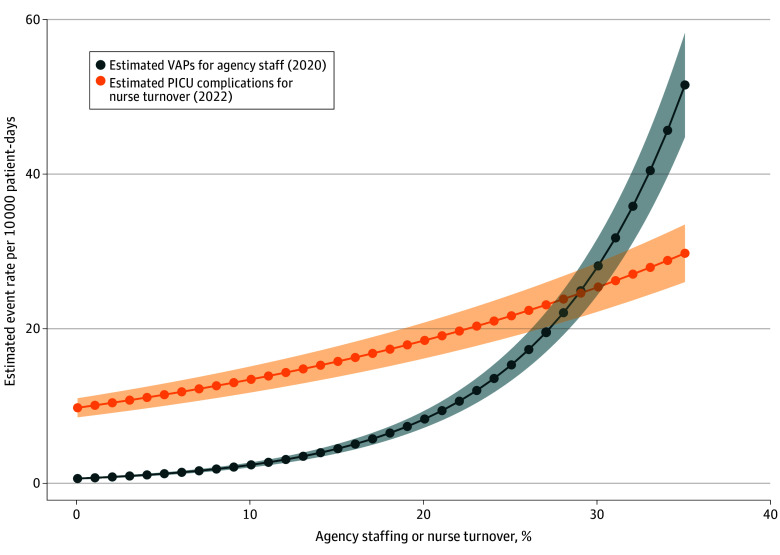
Estimated Outcomes Based on Modeling for Agency Nurses in 2020 and Nursing Turnover in 2022 PICU indicates pediatric intensive care unit; VAP, ventilator-associated pneumonia. Lines represent the respective mean percentage for the study year, and the bands represent the 95% CIs.

### Changes in Agency Staffing During the Public Health Emergency and Outcomes

Of the 20 PICUs, 10 experienced variability (>10% change from 2019 baseline) in the proportion of agency nurses compared with the prepandemic 2019 baseline staffing metrics (eTables 20 and 21 in Supplement 1). Variations in staffing measures during the study years were complex, as illustrated in the alluvial diagrams in the eFigure in Supplement 1. PICUs with variability in the proportion of agency staff also experienced greater nurse turnover compared with PICUs with a stable proportion of agency staff (15.9% [IQR, 13.0%-19.0%] vs 14.9% [11.1%-17.6%] nurse turnover; *P* < .001). More patients experienced PICU complications in those with a variable proportion of agency staff (9695 of 120 017 [8.1%]) compared with a stable proportion of agency staff (7141 of 98 772 [7.2%]) (odds ratio, 1.06; 95% CI, 1.03-1.09; *P* < .001). Median LOS was shorter in PICUs with a variable proportion of agency staff, and there were fewer patients who experienced cardiac arrest and who required RRT.

## Discussion

In this large, cross-sectional linked database study, we identified a complex pattern of associations between PICU nurse staffing measures and patient outcomes during the COVID-19 pandemic. Pressures on the health care system during COVID-19 resulted in significant nursing turnover and an increased reliance on incorporating a higher proportion of agency nurses. Instability in staffing, as evident by a substantial fluctuation in the proportion of agency nurse staff, was variably associated with more care-related complications. Periods with higher proportions of agency staff nurses and increased nursing turnover were associated with an increase in PICU complications and mortality, although the findings were not consistent across all years. However, the identified possible associations between nurse staffing and PICU complications after adjusting for patient illness severity and controlling for hospital effects suggest both a plausible and clinically meaningful impact of modifiable components of nurse staffing on important patient outcomes.

A qualitative assessment of nurses’ experiences during the pandemic^32^ identified new protocols, increased workloads, and an ostensible lack of adequate preparation by health systems as stressors contributing to many nurses leaving bedside acute care positions, or in some cases exiting the workforce altogether. Alterations in typical societal functions, such as nationwide lockdowns that limited options for childcare, compounded with increased and novel workplace stressors, further depleted the usual nursing workforce.^33^ As noted by the National Academy of Medicine, temporary agency nurses are often less familiar with multiple key components of an organization, including the information systems, facility layout, standard pathways, ways of coordinating and managing work, and other key processes.^7^ A sudden surge in the need for agency nurse staff early in the pandemic may have served as an unprecedented stress test highlighting the need for health systems to rapidly develop and implement plans for mitigating the drawbacks of agency nurse staff while realizing their benefits. The nominal association we identified between the nurse turnover and PICU mortality in 2020, early in the pandemic, could represent the need for strategies to quickly incorporate agency nurses into standing workforces.

PICUs demonstrating variability in nurse staffing during the pandemic also had a significantly greater proportion of patients who experienced at least 1 complication of PICU care. Nurse staffing instability may be an important source of disruption to nurse team dynamics during the pandemic, as has been shown in prior nursing literature about nursing turnover’s effect on outcomes.^34^ In many cases, sudden and substantial increases in temporary agency nurses were accompanied by related challenges to established nurse teams, including loss of senior nurse experience, young graduates entering high-acuity environments, wavering trust in health care hierarchies, and compromised psychological safety.^35^ In the aftermath of the pandemic, reduced psychological safety has been identified as a substantial contributor to nurse burnout with negative influences on nurse retention and team performance.^36,37,38^ Health care environments with high performing teams have better patient outcomes than those with suboptimal teamwork.^39^ Nurses serve as a vital component of improved care-related outcomes, and the importance of creating a positive work environment for them cannot be understated.^40^ The degree to which agency nurse staff were detrimental, beneficial, or simply a representative surrogate of other independent forces in health care early in the COVID-19 pandemic warrants further study. Our findings suggest the need to better understand the relationships among nurse staffing measures, team dynamics, and patient outcomes.

We observed an association between health care–associated infections and nurse staffing measures. Nurse burnout and experience have complex associations with acquired infections.^40,41,42,43^ A large administrative data study identified possible links among nurse staffing, nurse experience, and health care–acquired infections, although the relationships appeared inconsistent.^41^ Similarly, we found an association between measured staffing indicators and patient-centered outcomes, likely reflecting unaccounted risk factors in the relationship of nurse staffing with patient outcomes. For example, nurse inexperience can be offset by effective mentors^44^ and supportive environments attuned to the needs of novice learners.^45^ A Cochrane systematic review of hospital nurse-staffing models and patient outcomes^44^ noted the relationship between staffing and nonmortality outcomes to be unclear, citing the need for high-quality study designs, such as prospective, randomized clinical trials and rigorous interrupted time series analyses. Our work adds to a body of literature that highlights the need for scaled, multicenter evaluation of staffing models and retention of experienced nurses to better clarify interactions with patient outcomes.

### Limitations

Limitations of the present study include the inability to completely account for differences in unit characteristics, onboarding programs, individual nurse experience, staffing ratios, physician experience, and acute complexity of patient illness. These risk factors likely interact with nurse staffing measures and patient outcomes. While we were unable to measure these risk factors in our study, the identified associations between nurse staffing patterns and measures are plausible and warrant more detailed evaluation. The list of PICU complications is broad, and it is difficult to completely disentangle the direct impact of nursing practices on all complications. Within the available data, the variable associations among nurse turnover, agency nurses, and the outcomes within the study could represent system strain rather than direct nurse practices. While we had access to patient-specific demographic and outcome measures, the human resources measures were only available at the unit level and on a yearly basis. More detailed data on patient assignments and the skill-mix, training, and experience level differences between agency nurses and full-time bedside nurses would have enriched the analyses.^40^ Variability in patient volume and disease processes during the COVID-19 pandemic have been well-described^46,47^; accordingly, our analysis of the incidence of yearly complications may overlook more nuanced temporal associations among staffing structures, patient populations, and outcomes. While nursing turnover and agency use varied during the pandemic, they occurred amid broader strain on the health care system. Staffing shortages among respiratory therapists^48^ and pharmacy technicians,^49^ among other currently unmeasured forces, also may have plausibly contributed to patient outcomes.

## Conclusions

In this cross-sectional study evaluating the association of nurse staffing measures with PICU outcomes during the COVID-19 pandemic, our findings suggest that an increased proportion of agency nurse staffing in PICUs exhibited complex associations with increased PICU complications and adverse patient outcomes. These results should be used to guide the development of future, prospective studies aimed at interrogating the relationship between nurse staffing and patient outcomes, with the goal of understanding and implementing optimal staffing practices for the high-stakes field of pediatric intensive care medicine.

## Notes

Supplement 1. **eMethods.** Agency Grouping, Turnover Grouping, Variability, and Analysis **eTable 1.** Composite PICU Complication List **eTable 2.** Patient Demographics Among Agency Groups 2019 **eTable 3.** Outcomes by the Proportion of Agency Nurse Staff for 2019 **eTable 4.** Patient Demographics Among Agency Groups 2020 **eTable 5.** Outcomes by the Proportion of Agency Nurse Staff for 2020 **eTable 6.** Patient Demographics Among Agency Groups 2021 **eTable 7.** Outcomes by the Proportion of Agency Nurse Staff for 2021 **eTable 8.** Patient Demographics Among Agency Groups 2022 **eTable 9.** Outcomes by the Proportion of Agency Nurse Staff for 2022 **eTable 10.** Demographics Among Groups Defined by Nurse Turnover **eTable 11.** Outcomes Defined by Nurse Turnover **eTable 12.** Patient Demographics Among Nursing Groups 2019 **eTable 13.** Patient Outcomes Among Nursing Groups 2019 **eTable 14.** Patient Demographics Among Nursing Groups 2020 **eTable 15.** Patient Outcomes Among Nursing Groups 2020 **eTable 16.** Patient Demographics Among Nursing Groups 2021 **eTable 17.** Patient Outcomes Among Nursing Groups 2021 **eTable 18.** Patient Demographics Among Nursing Groups 2022 **eTable 19.** Patient Outcomes Among Nursing Groups 2022 **eTable 20.** Demographics Among Units With Stable or Variable Agency Staffing Between 2019 and 2022 **eTable 21.** Outcomes Among Units With Stable or Variable Agency Staffing Between 2019 and 2022 **eFigure.** Alluvial Diagrams for Hospital Proportion Agency Staff and Nurse Turnover

Supplement 2.
Data Sharing Statement

## References

[1] American Hospital Association. Data brief: health care workforce challenges threaten hospitals’ ability to care for patients. Accessed October 20, 2023. https://www.aha.org/fact-sheets/2021-11-01-data-brief-health-care-workforce-challenges-threaten-hospitals-ability-care

[2] Myers LC, Liu VX. The COVID-19 pandemic strikes again and again and again. JAMA Netw Open. 2022;5(3):e221760. doi:10.1001/jamanetworkopen.2022.1760 35262720

[3] American Organization for Nursing Leadership. Nursing leadership insight longitudinal study. April 4, 2024. Accessed April 4, 2024. https://www.aonl.org/resources/nursing-leadership-survey

[4] Yang YT, Mason DJ. COVID-19’s impact on nursing shortages, the rise of travel nurses, and price gouging. Health Affairs Forefront. Published online January 28, 2022. doi:10.1377/forefront.20220125.695159

[5] Chervoni-Knapp T. The staffing shortage pandemic. J Radiol Nurs. 2022;41(2):74-75. doi:10.1016/j.jradnu.2022.02.007 35431684 PMC8989263

[6] Carrazana C. Travel nurses saw an increase in pay during the pandemic. Now, they could lose those benefits. J Adv Pract Nurs. February 15, 2022. Accessed September 10, 2024. https://www.asrn.org/journal-advanced-practice-nursing/2708-travel-nurses-saw-an-increase-in-pay-during-the-pandemic-now-they-could-lose-those-benefits.html

[7] Institute of Medicine (US) Committee on the Work Environment for Nurses and Patient Safety; Page A, ed. Keeping Patients Safe: Transforming the Work Environment of Nurses. National Academies Press; 2004. 25009849

[8] Page AEK. Temporary, agency, and other contingent workers. In: Hughes RG, ed. Patient Safety and Quality: An Evidence-Based Handbook for Nurses. Advances in Patient Safety. Agency for Healthcare Research and Quality; 2008. 21328752

[9] Vander Weerdt C, Peck JA, Porter T. Travel nurses and patient outcomes: a systematic review. Health Care Manage Rev. 2023;48(4):352-362. doi:10.1097/HMR.0000000000000383 37615945

[10] Shin S, Park JH, Bae SH. Nurse staffing and nurse outcomes: a systematic review and meta-analysis. Nurs Outlook. 2018;66(3):273-282. doi:10.1016/j.outlook.2017.12.002 29685321

[11] Aiken LH, Clarke SP, Sloane DM, Sochalski J, Silber JH. Hospital nurse staffing and patient mortality, nurse burnout, and job dissatisfaction. JAMA. 2002;288(16):1987-1993. doi:10.1001/jama.288.16.1987 12387650

[12] Lasater KB, Aiken LH, Sloane D, . Patient outcomes and cost savings associated with hospital safe nurse staffing legislation: an observational study. BMJ Open. 2021;11(12):e052899. doi:10.1136/bmjopen-2021-052899 34880022 PMC8655582

[13] Griffiths P, Ball J, Drennan J, . Nurse staffing and patient outcomes: strengths and limitations of the evidence to inform policy and practice: a review and discussion paper based on evidence reviewed for the National Institute for Health and Care Excellence Safe Staffing guideline development. Int J Nurs Stud. 2016;63:213-225. doi:10.1016/j.ijnurstu.2016.03.012 27130150

[14] Dall’Ora C, Saville C, Rubbo B, Turner L, Jones J, Griffiths P. Nurse staffing levels and patient outcomes: a systematic review of longitudinal studies. Int J Nurs Stud. 2022;134:104311. doi:10.1016/j.ijnurstu.2022.104311 35780608

[15] Shekelle PG. Nurse-patient ratios as a patient safety strategy: a systematic review. Ann Intern Med. 2013;158(5 pt 2):404-409. doi:10.7326/0003-4819-158-5-201303051-00007 23460097

[16] Jansson MM, Syrjälä HP, Ala-Kokko TI. Association of nurse staffing and nursing workload with ventilator-associated pneumonia and mortality: a prospective, single-center cohort study. J Hosp Infect. 2019;101(3):257-263. doi:10.1016/j.jhin.2018.12.001 30529704

[17] Hugonnet S, Uçkay I, Pittet D. Staffing level: a determinant of late-onset ventilator-associated pneumonia. Crit Care. 2007;11(4):R80. doi:10.1186/cc5974 17640384 PMC2206525

[18] Shang J, Needleman J, Liu J, Larson E, Stone PW. Nurse staffing and healthcare-associated infection, unit-level analysis. J Nurs Adm. 2019;49(5):260-265. doi:10.1097/NNA.0000000000000748 31008835 PMC6478399

[19] Van T, Annis AM, Yosef M, . Nurse staffing and healthcare-associated infections in a national healthcare system that implemented a nurse staffing directive: multi-level interrupted time series analyses. Int J Nurs Stud. 2020;104:103531. doi:10.1016/j.ijnurstu.2020.103531 32062053

[20] Twigg DE, Kutzer Y, Jacob E, Seaman K. A quantitative systematic review of the association between nurse skill mix and nursing-sensitive patient outcomes in the acute care setting. J Adv Nurs. 2019;75(12):3404-3423. doi:10.1111/jan.14194 31483509 PMC6899638

[21] Needleman J, Buerhaus P, Mattke S, Stewart M, Zelevinsky K. Nurse-staffing levels and the quality of care in hospitals. N Engl J Med. 2002;346(22):1715-1722. doi:10.1056/NEJMsa012247 12037152

[22] Driscoll A, Grant MJ, Carroll D, . The effect of nurse-to-patient ratios on nurse-sensitive patient outcomes in acute specialist units: a systematic review and meta-analysis. Eur J Cardiovasc Nurs. 2018;17(1):6-22. doi:10.1177/1474515117721561 28718658

[23] Kane RL, Shamliyan TA, Mueller C, Duval S, Wilt TJ. The association of registered nurse staffing levels and patient outcomes: systematic review and meta-analysis. Med Care. 2007;45(12):1195-1204. doi:10.1097/MLR.0b013e3181468ca3 18007170

[24] Marcin JP, Rutan E, Rapetti PM, Brown JP, Rahnamayi R, Pretzlaff RK. Nurse staffing and unplanned extubation in the pediatric intensive care unit. Pediatr Crit Care Med. 2005;6(3):254-257. doi:10.1097/01.PCC.0000160593.75409.6B 15857520

[25] Estabrooks CA, Midodzi WK, Cummings GG, Ricker KL, Giovannetti P. The impact of hospital nursing characteristics on 30-day mortality. Nurs Res. 2005;54(2):74-84. doi:10.1097/00006199-200503000-00002 15778649

[26] Rabinowicz S, Leshem E, Pessach IM. COVID-19 in the pediatric population-review and current evidence. Curr Infect Dis Rep. 2020;22(11):29. doi:10.1007/s11908-020-00739-6 32982599 PMC7501762

[27] Be’er M, Amirav I, Cahal M, . Unforeseen changes in seasonality of pediatric respiratory illnesses during the first COVID-19 pandemic year. Pediatr Pulmonol. 2022;57(6):1425-1431. doi:10.1002/ppul.25896 35307986 PMC9088630

[28] Solventum All Patient Refined Diagnosis Related Groups (APR DRGs) classification system. Accessed April 4, 2024. https://www.solventum.com/en-us/home/health-information-technology/solutions/apr-drg/

[29] Pediatric Health Information System—Pediatric Analytic Solutions. Children’s Hospital Association. Accessed June 15, 2022. https://www.childrenshospitals.org/

[30] Green JA. Too many zeros and/or highly skewed? a tutorial on modelling health behaviour as count data with Poisson and negative binomial regression. Health Psychol Behav Med. 2021;9(1):436-455. doi:10.1080/21642850.2021.1920416 34104569 PMC8159206

[31] UCLA Advanced Research Computing. Negative binomial regression Stata annotated output. Accessed July 23, 2024. https://stats.oarc.ucla.edu/stata/output/negative-binomial-regression/

[32] Kelley MM, Zadvinskis IM, Miller PS, . United States nurses’ experiences during the COVID-19 pandemic: a grounded theory. J Clin Nurs. 2022;31(15-16):2167-2180. doi:10.1111/jocn.16032 34606133

[33] Cantor J, Whaley CM, Ward J, Jena AB. COVID-19 school closures were associated with a decline in employment for female nurses with young children. Health Aff (Millwood). 2024;43(9):1329-1337. doi:10.1377/hlthaff.2023.01250 39226495 PMC12224026

[34] Shen K, McGarry BE, Gandhi AD. Health care staff turnover and quality of care at nursing homes. JAMA Intern Med. 2023;183(11):1247-1254. doi:10.1001/jamainternmed.2023.5225 37812410 PMC10562988

[35] Sherman RO. Rebuilding your nursing team in 2022. Nurse Lead. 2022;20(1):14-15. doi:10.1016/j.mnl.2021.10.007 34785990 PMC8586900

[36] Dale-Tam J. Why establishing psychological safety is crucial while teaching in a post-pandemic health-care setting. March 18, 2024. Accessed December 2, 2024. https://www.canadian-nurse.com/blogs/cn-content/2024/03/18/psychological-safety-teaching-post-pandemic

[37] Ma Y, Faraz NA, Ahmed F, . Curbing nurses’ burnout during COVID-19: the roles of servant leadership and psychological safety. J Nurs Manag. 2021;29(8):2383-2391. doi:10.1111/jonm.13414 34259372 PMC8420609

[38] Johnson C, Marcus-Aiyeku U, Hessels A. Low levels of psychological safety in inpatient medical-surgical nurses on the tail end the COVID-19 pandemic. Antimicrob Steward Healthc Epidemiol. 2024;4(S1):s129. doi:10.1017/ash.2024.292

[39] Rosen MA, DiazGranados D, Dietz AS, . Teamwork in healthcare: key discoveries enabling safer, high-quality care. Am Psychol. 2018;73(4):433-450. doi:10.1037/amp0000298 29792459 PMC6361117

[40] Butler GA, Hupp DS. Pediatric quality and safety: a nursing perspective. Pediatr Clin North Am. 2016;63(2):329-339. doi:10.1016/j.pcl.2015.11.005 27017039

[41] Cimiotti JP, Aiken LH, Sloane DM, Wu ES. Nurse staffing, burnout, and health care-associated infection. Am J Infect Control. 2012;40(6):486-490. doi:10.1016/j.ajic.2012.02.029 22854376 PMC3509207

[42] Peutere L, Terho K, Pentti J, . Nurse staffing level, length of work experience, and risk of health care–associated infections among hospital patients: a prospective record linkage study. Med Care. 2023;61(5):279-287. doi:10.1097/MLR.0000000000001843 36939226 PMC10079297

[43] Alanazi FK, Lapkin S, Molloy L, Sim J. Healthcare-associated infections in adult intensive care units: a multisource study examining nurses’ safety attitudes, quality of care, missed care, and nurse staffing. Intensive Crit Care Nurs. 2023;78:103480. doi:10.1016/j.iccn.2023.103480 37379679

[44] Butler M, Schultz TJ, Halligan P, . Hospital nurse-staffing models and patient- and staff-related outcomes. Cochrane Database Syst Rev. 2019;4(4):CD007019. doi:10.1002/14651858.CD007019.pub3 31012954 PMC6478038

[45] Ferguson LM. From the perspective of new nurses: what do effective mentors look like in practice? Nurse Educ Pract. 2011;11(2):119-123. doi:10.1016/j.nepr.2010.11.003 21159558

[46] Pelletier JH, Rakkar J, Au AK, Fuhrman D, Clark RSB, Horvat CM. Trends in US pediatric hospital admissions in 2020 compared with the decade before the COVID-19 pandemic. JAMA Netw Open. 2021;4(2):e2037227. doi:10.1001/jamanetworkopen.2020.37227 33576819 PMC7881361

[47] Ramgopal S, Pelletier JH, Rakkar J, Horvat CM. Forecast modeling to identify changes in pediatric emergency department utilization during the COVID-19 pandemic. Am J Emerg Med. 2021;49:142-147. doi:10.1016/j.ajem.2021.05.047 34111834 PMC8555971

[48] Fleming K, George JL, Bazelak SJ, . Optimizing respiratory therapy resources by de-implementing low-value care. Respir Care. 2023;68(5):559-564. doi:10.4187/respcare.10712 37015815 PMC10171347

[49] ASHP. Hospitals and health systems experiencing severe shortage of pharmacy technicians. March 15, 2022. Accessed March 24, 2025. https://news.ashp.org/News/ashp-news/2022/03/15/hospitals-and-health-systems-experiencing-severe-shortage-of-pharmacy-technicians

