# Error in the Byline

**DOI:** 10.1001/jamanetworkopen.2025.31887

**Published:** 2025-08-15

In the Original Investigation titled “Pediatric Intensive Care Nurse Staffing Measures and Patient Outcomes During the COVID-19 Pandemic,” published on June 12, 2025, Patrick M. Kochanek’s middle initial was incorrect. This article has been corrected.^1^

## References

[1] Taylor WM, Pelletier J, Heneghan JA, . Pediatric intensive care nurse staffing measures and patient outcomes during the COVID-19 pandemic. JAMA Netw Open. 2025;8(6):e2515376. doi:10.1001/jamanetworkopen.2025.15376 40504525 PMC12163677

