# Supplementary material for: Pediatric Intensive Care Nurse Staffing Measures and Patient Outcomes During the COVID-19 Pandemic
Source: JAMA Netw Open. 2025 Jun 12;8(6):e2515376. doi: 10.1001/jamanetworkopen.2025.15376 (PMC12163677; doi:10.1001/jamanetworkopen.2025.15376)
Supplement: Supplement 1. — eMethods. Agency Grouping, Turnover Grouping, Variability, and Analysis eTable 1. Composite PICU Complication List eTable 2. Patient Demographics Among Agency Groups 2019 eTable 3. Outcomes by the Proportion of Agency Nurse Staff for 2019 eTable 4. Patient Demographics Among Agency Groups 2020 eTable 5. Outcomes by the Proportion of Agency Nurse Staff for 2020 eTable 6. Patient Demographics Among Agency Groups 2021 eTable 7. Outcomes by the Proportion of Agency Nurse Staff for 2021 eTable 8. Patient Demographics Among Agency Groups 2022 eTable 9. Outcomes by the Proportion of Agency Nurse Staff for 2022 eTable 10. Demographics Among Groups Defined by Nurse Turnover eTable 11. Outcomes Defined by Nurse Turnover eTable 12. Patient Demographics Among Nursing Groups 2019 eTable 13. Patient Outcomes Among Nursing Groups 2019 eTable 14. Patient Demographics Among Nursing Groups 2020 eTable 15. Patient Outcomes Among Nursing Groups 2020 eTable 16. Patient Demographics Among Nursing Groups 2021 eTable 17. Patient Outcomes Among Nursing Groups 2021 eTable 18. Patient Demographics Among Nursing Groups 2022 eTable 19. Patient Outcomes Among Nursing Groups 2022 eTable 20. Demographics Among Units With Stable or Variable Agency Staffing Between 2019 and 2022 eTable 21. Outcomes Among Units With Stable or Variable Agency Staffing Between 2019 and 2022 eFigure. Alluvial Diagrams for Hospital Proportion Agency Staff and Nurse Turnover [file jamanetwopen-e2515376-s001.pdf]

Supplementary Online Content

Taylor WM, Pelletier J, Heneghan JA, et al. Pediatric intensive care nurse staffing measures and patient outcomes during the COVID-19 pandemic. *JAMA Netw Open.* 2025;8(6):e2515376. doi:10.1001/jamanetworkopen.2025.15376

eMethods. Agency Grouping, Turnover Grouping, Variability, and Analysis

eTable 1. Composite PICU Complication List

eTable 2. Patient Demographics Among Agency Groups 2019

eTable 3. Outcomes by the Proportion of Agency Nurse Staff for 2019

eTable 4. Patient Demographics Among Agency Groups 2020

eTable 5. Outcomes by the Proportion of Agency Nurse Staff for 2020

eTable 6. Patient Demographics Among Agency Groups 2021

eTable 7. Outcomes by the Proportion of Agency Nurse Staff for 2021

eTable 8. Patient Demographics Among Agency Groups 2022

eTable 9. Outcomes by the Proportion of Agency Nurse Staff for 2022

eTable 10. Demographics Among Groups Defined by Nurse Turnover

eTable 11. Outcomes Defined by Nurse Turnover

eTable 12. Patient Demographics Among Nursing Groups 2019

eTable 13. Patient Outcomes Among Nursing Groups 2019

eTable 14. Patient Demographics Among Nursing Groups 2020

eTable 15. Patient Outcomes Among Nursing Groups 2020

eTable 16. Patient Demographics Among Nursing Groups 2021

eTable 17. Patient Outcomes Among Nursing Groups 2021

eTable 18. Patient Demographics Among Nursing Groups 2022

eTable 19. Patient Outcomes Among Nursing Groups 2022

eTable 20. Demographics Among Units With Stable or Variable Agency Staffing Between 2019 and 2022

eTable 21. Outcomes Among Units With Stable or Variable Agency Staffing Between 2019 and 2022

eFigure. Alluvial Diagrams for Hospital Proportion Agency Staff and Nurse Turnover

This supplementary material has been provided by the authors to give readers additional information about their work.

© 2025 Taylor WM et al. *JAMA Network Open*.

eMethods. Agency Grouping, Turnover Grouping, Variability, and Analysis

Agency Grouping

PICUs were grouped into agency groups based off manual sorting using the histogram below. These groups were established as the baseline values in 2019 and utilized as the same group definitions throughout the study years. The groups were defined as: Group 1 had 0.0% agency nursing, Group 2 0.1-5.0%, Group 3 5.1-10%, and Group 4 >10.0% agency staffing per year.

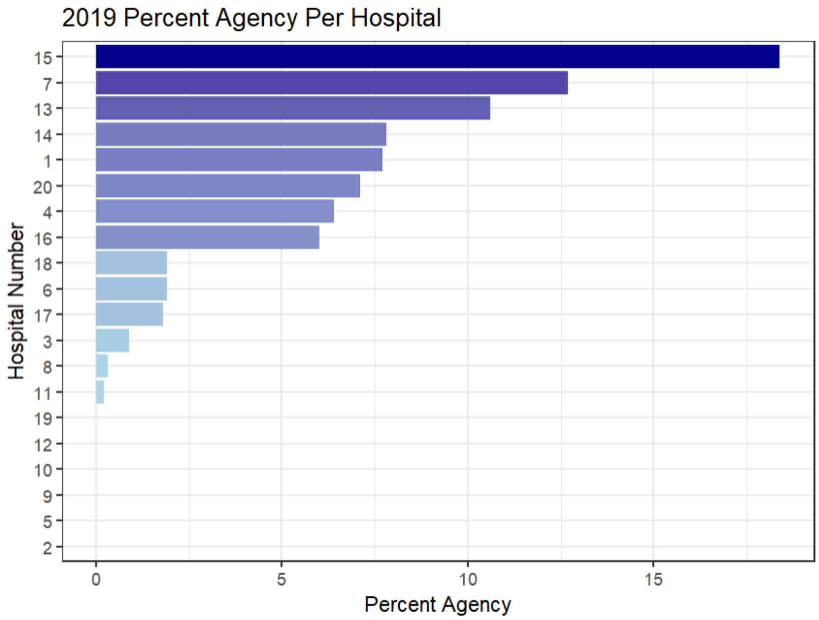

Nurse Turnover Grouping

PICUS were grouped into nurse turnover groups based on manual sorting of the histogram below. These groups were calculated by the mean nurse turnover throughout the study years at all hospitals. The groups were defined as: Group 1 <12.5% nurse turnover, Group 2 ≥12.5% and <15.0%, Group 3 ≥15.0% and <17.5%, and Group 4 ≥17.5% nurse turnover.

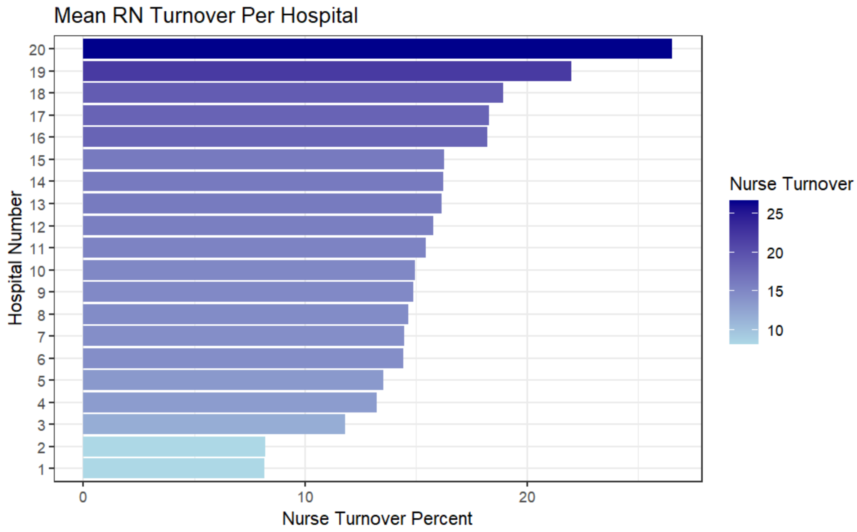

© 2025 Taylor WM et al. *JAMA Network Open*.

Agency Variability Definition

We found no data within prior nurse staffing research to specifically define agency variability. Agency variability and stable agency were therefore derived manually based off the mean percent difference in agency staffing across the years. The mean difference across study all hospitals, with the baseline agency staff levels defined as 2019, was 0.3% with a standard deviation of 7.48%. To conservatively define agency variability, we chose 10% as the cutoff, outside the standard deviation within the cohort.

Negative Binomial Exposure Offset

Regression methods for count-based outcomes necessitate adjustment for an exposure variable. Its use facilitates comparison of the rate of events across groups, in our study PICU complications or mortality. [1] The log offset norms the exposure variable, days for PICU complications and admissions for mortality, across study hospitals as a patient admitted for longer has a greater time frame to experience an PICU complication. Similarly, hospitals with more patient admissions may have higher numbers of death. Only patients on ventilators were at risk for a ventilator associated pneumonia and their exposure was theoretically less than total length of stay. We ran the ventilator associated pneumonia model using ventilator days as the exposure variable. Ventilator days was compiled using billed ventilator days submitted to PHIS. Only encounters with the mechanical ventilator flag of "Yes" and having billed ventilator days were included in the VAP model. There were 65,043 patients (29.7%) of study patients who were flagged for mechanical ventilation. Of those mechanically ventilated, 2837 (4.4%) had no ventilator day total (i.e., didn't have billing data for ventilator days).

[1] Meyer J. The Importance of Including an Exposure Variable in Count Models. The Analysis Factor. November 19, 2020. Accessed October 3, 2024. https://www.theanalysisfactor.com/including-an-exposure-variable-in-count-models/

© 2025 Taylor WM et al. *JAMA Network Open*.

eTable 1. Composite PICU Complication List

Surgical Complications in PHIS and Count	
I9581 - Postprocedural Hypotension	1082
J9502 - Infection Of Tracheostomy Stoma	253
J9509 - Other Tracheostomy Complication	1070
J95811 - Postprocedural Pneumothorax	598
J95821 - Acute Postprocedural Respiratory Failure	3490
K9402 - Colostomy Infection	1
K9412 - Enterostomy Infection	37
K9422 - Gastrostomy Infection	461
K9429 - Other Complications Of Gastrostomy	778
T80211A - Bloodstream Infection Due To Central Venous Catheter, Init	1865
T80212A - Local Infection Due To Central Venous Catheter, Init Encntr	97
T8141XA - Infct Fol A Proc, Superfic Incisional Surgical Site, Init	463
T8149XA - Infection Following A Procedure, Other Surgical Site, Init	291
T82118A - Breakdown (mechanical) Of Cardiac Electronic Device, Init	28
T82120A - Displacement Of Cardiac Electrode, Initial Encounter	12
T82190A - Mech Compl Of Cardiac Electrode, Initial Encounter	56
T82524A - Displacement Of Infusion Catheter, Initial Encounter	382
T83028A - Displacement Of Other Urinary Catheter, Initial Encounter	12
T83511A - I/i React D/t Indwelling Urethral Catheter, Init	85
T83518A - I/i React D/t Other Urinary Catheter, Initial Encounter	84
**Medical Complications in PHIS and Count**	
D6951 - Posttransfusion Purpura	9
D84821 - Immunodeficiency Due To Drugs	3607
E8771 - Transfusion Associated Circulatory Overload	77
I953 - Hypotension Of Hemodialysis	106
J954 - Chemical Pneumonitis Due To Anesthesia	10
J9584 - Transfusion-related Acute Lung Injury (trali)	66
J95850 - Mechanical Complication Of Respirator	38
J95851 - Ventilator Associated Pneumonia	1341
J95859 - Other Complication Of Respirator [ventilator]	40
M96A3 - Mult Fx Of Ribs Assoc W Chest Comprsn And Cardiopulm Resus	2
R5084 - Febrile Nonhemolytic Transfusion Reaction	96
T383X5A - Adverse Effect Of Insulin And Oral Hypoglycemic Drugs, Init	49
T410X5A - Adverse Effect Of Inhaled Anesthetics, Initial Encounter	47
T411X5A - Adverse Effect Of Intravenous Anesthetics, Initial Encounter	37
T41205A - Adverse Effect Of Unsp General Anesthetics, Init Encntr	45
T41295A - Adverse Effect Of Other General Anesthetics, Init Encntr	177
T413X5A - Adverse Effect Of Local Anesthetics, Initial Encounter	37
T4145XA - Adverse Effect Of Unspecified Anesthetic, Initial Encounter	122
T50905A - Adverse Effect Of Unsp Drug/meds/biol Subst, Init	243

© 2025 Taylor WM et al. *JAMA Network Open*.

T50995A - Adverse Effect Of Drug/meds/biol Subst, Init	354
T782XXA - Anaphylactic Shock, Unspecified, Initial Encounter	171
T783XXA - Angioneurotic Edema, Initial Encounter	152
T800XXA - Air Embolism Fol Infusion, Tranfs And Theraputc Inject, Init	14
T801XXA - Vascular Comp Fol Infusn, Tranfs And Theraputc Inject, Init	62
T8029XA - Infct Fol Oth Infusion, Transfuse And Theraputc Inject, Init	46
T80319A - Abo Incompatibility W Hemolytic Transfs React, Unsp, Init	2
T8040XA - Rh Incompat React Due To Tranfs Of Bld/bld Prod, Unsp, Init	1
T8051XA - Anaphylactic Reaction Due To Admin Blood/products, Init	43
T8052XA - Anaphylactic Reaction Due To Vaccination, Initial Encounter	2
T8059XA - Anaphylactic Reaction Due To Other Serum, Initial Encounter	8
T8061XA - Oth Serum Reaction Due To Admin Blood/products, Init	32
T8069XA - Other Serum Reaction Due To Other Serum, Initial Encounter	31
T80810A - Extravasation Of Vesicant Antineoplastic Chemotherapy, Init	2
T80818A - Extravasation Of Other Vesicant Agent, Initial Encounter	333
T8082XA - Complication Of Immune Effector Cellular Therapy, Init	14
T8089XA - Oth Comp Fol Infusion, Transfuse And Theraputc Inject, Init	1144
T80910A - Acute Hemolytic Transfs React, Unsp Incompatibility, Init	5
T80911A - Delayed Hemolytic Transfs React, Unsp Incompatibility, Init	4
T80919A - Hemolytic Transfs React, Unsp Incompat, Unsp Ac/delay, Init	2
T8092XA - Unspecified Transfusion Reaction, Initial Encounter	128
T8181XA - Complication Of Inhalation Therapy, Initial Encounter	3
T880XXA - Infection Following Immunization, Initial Encounter	5
T881XXA - Oth Complications Following Immunization, Nec, Init	17
T882XXA - Shock Due To Anesthesia, Initial Encounter	8
T883XXA - Malignant Hyperthermia Due To Anesthesia, Initial Encounter	10
T884XXA - Failed Or Difficult Intubation, Initial Encounter	660
T8851XA - Hypothermia Following Anesthesia, Initial Encounter	14
T8859XA - Other Complications Of Anesthesia, Initial Encounter	81
T886XXA - Anaphyl Reaction Due To Advrs Eff Drug/med Prop Admin, Init	314
T887XXA - Unsp Adverse Effect Of Drug Or Medicament, Init Encntr	9
T888XXA - Oth Complications Of Surgical And Medical Care, Nec, Init	67
T889XXA - Complication Of Surgical And Medical Care, Unsp, Init Encntr	1

© 2025 Taylor WM et al. *JAMA Network Open*.

Table 2. Patient Demographics Among Agency Groups 2019

Characteristic[1]	Overall	Group 1	Group 2	Group 3	Group 4
**N**	61,289	5,048	28,475	11,460	16,306
Number of PICUs	20	6	6	5	3
Proportion of agency nurse staff, %	--	0	0.1-5.0	5.1-10	>10
Admit age[a]	40 (8-129)	41 (8-133)	35 (7-121)	44 (9-131)	46 (11-137)
Gender[b]					
Female	27,704 (45)	2,307 (46)	12,736 (45)	5,083 (44)	7,578 (46)
Male	33,556 (55)	2,741 (54)	15,727 (55)	6,361 (56)	8,727 (54)
Unspecified	29 (<0.1)	0 (0)	12 (<0.1)	16 (0.1)	1 (<0.1)
Complex chronic condition[b]	38,937 (64)	2,903 (58)	18,699 (66)	7,558 (66)	9,777 (60)
Race[b]					
Asian	2,637 (4.3)	115 (2.3)	1,010 (3.5)	502 (4.4)	1,010 (6.2)
American Indian	300 (0.5)	23 (0.5)	86 (0.3)	59 (0.5)	132 (0.8)
Black	11,707 (19)	1,000 (20)	7,013 (25)	2,591 (23)	1,103 (6.8)
Pacific Islander	418 (0.7)	10 (0.2)	70 (0.2)	108 (0.9)	230 (1.4)
White	37,201 (61)	3,625 (72)	15,984 (56)	6,899 (60)	10,693 (66)
Other Race	6,797 (11)	124 (2.5)	2,240 (7.9)	1,242 (11)	3,191 (20)
% nurse turnover[a]	13.0 (10.5-14.9)	12.8 (12.8-12.8)	11.1 (10.4-14.9)	12.1 (12.1- 16.1)	13.4 (13.4-14.7)
Severity level[c]	2.86 (0.99)	2.73 (1.00)	2.93 (0.95)	3.01 (0.96)	2.68 (1.02)
Expected length of stay[a]	5 (3-11)	5 (3-9)	6 (3-12)	6 (3-12)	4 (3-8)

[a] median (IQR); [b] n (%); [c] mean (SD)

eTable 3. Outcomes by the Proportion of Agency Nurse Staff for 2019

Characteristic[1]	Overall	Group 1	Group 2	Group 3	Group 4	*P*
**N**	61,289	5,408	28,475	11,460	16,306	---
Number of PICUs	20	6	6	5	3	---
Length of stay[a]	12 (2-10)	11 (3-10)	13 (3-11)	12 (3-11)	9 (2-8)	<0.001[c]
Length of stay ratio (IQR)	0.90 (0.55-1.41)	0.98 (0.62-1.55)	0.93 (0.58-1.44)	0.85 (0.49-1.38)	0.84 (0.51-1.33)	<0.001[c]
PICU days[a]	2 (1-5)	2 (1-4)	2 (1-5)	2 (1-4)	2 (1-4)	<0.001[c]
Patients with PICU complication[b]	3,905 (6.4)	251 (5.0)	1,979 (6.9)	854 (7.5)	821 (5.0)	<0.001[d]
PICU complications	4,384	304	2,515	1,014	1,001	---
CLABSIs[b]	447 (0.7)	48 (1.0)	204 (0.7)	82 (0.7)	113 (0.7)	0.30[d]
VAPs[b]	180 (0.3)	10 (0.2)	56 (0.2)	53 (0.5)	61 (0.4)	<0.001[d]
Cardiac arrest[b]	395 (0.6)	35 (0.7)	178 (0.6)	91 (0.8)	91 (0.6)	0.10[d]
Renal replacement therapy[b]	871 (1.4)	63 (1.2)	387 (1.4)	258 (2.3)	163 (1.0)	<0.001[d]
Vasoactive infusion[b]	15,288 (25)	1,444 (29)	7,476 (26)	3,517 (31)	2,851 (17)	<0.001[d]
ECMO[b]	584 (1.0)	50 (1.0)	261 (0.9)	135 (1.2)	138 (0.8)	0.052[d]
Mortality[b]	1,399 (2.3)	117 (2.3)	709 (2.5)	303 (2.6)	270 (1.7)	<0.001[d]

[a] mean (IQR); [b] n (%); [c] Kruskal-Wallis Rank Sum test; [d] Pearson Chi-squared test. Abbreviations: ECMO, extracorporeal membrane oxygenation; PICU, intensive care unit.

© 2025 Taylor WM et al. *JAMA Network Open*.

eTable 4. Patient Demographics Among Agency Groups 2020

Characteristic[1]	Overall	Group 1	2	3	4
N	48,337	4,035	22,021	8,862	13,419
Number of PICUs	20	6	9	3	2
Proportion of agency nurse staff, %	--	0	0.1-5.0	5.1-10	>10
Admit age (months)[a]	53 (10-148)	57 (11-149)	47 (8-144)	63 (13-153)	56 (11-149)
Gender[b]					
Female	22,085 (46)	1,858 (46)	10,117 (46)	3,998 (45)	6,112 (46)
Male	26,221 (54)	2,176 (54)	11,895 (54)	4,852 (55)	7,298 (54)
Unspecified	31 (<0.1)	1 (<0.1)	9 (<0.1)	12 (0.1)	9 (<0.1)
Complex chronic condition[b]	33,406 (69)	2,611 (65)	15,624 (71)	6,432 (73)	8,739 (65)
Race[b]					
Asian	1,984 (4.1)	91 (2.3)	741 (3.4)	394 (4.4)	758 (5.6)
American Indian	221 (0.5)	21 (0.5)	59 (0.3)	31 (0.3)	110 (0.8)
Black	9,269 (19)	775 (19)	5,506 (25)	2,081 (23)	907 (6.8)
Pacific Islander	268 (0.6)	7 (0.2)	29 (0.1)	81 (0.9)	151 (1.1)
White	29,406 (61)	2,913 (72)	12,591 (57)	5,348 (60)	8,554 (64)
Other Race	5,469 (11)	121 (3.0)	1,450 (6.6)	923 (10)	2,975 (22)
% nurse turnover[a]	12.4 (11.4-14.3)	13.9 (13.9-13.9)	11.5 (9.9-16.2)	12.4 (11.8-12.4)	12.5 (11.4-14.3)
Severity level[c]	2.93 (0.98)	2.86 (0.97)	2.98 (0.96)	3.11 (0.93)	2.75 (0.99)
Expected length of stay[a]	6 (3-12)	5 (3-12)	7 (3-12)	7 (3-12)	5 (3-9)

[a] median (IQR); [b] n (%); [c] mean (SD)

eTable 5. Outcomes by the Proportion of Agency Nurse Staff for 2020

Characteristic[1]	Overall	Group 1	2	3	4	*P*
N	48,337	4,035	22,021	8,862	13,419	---
Number of PICUs	20	6	9	3	2	---
Length of stay[a]	5 (2-11)	4 (2-10)	5 (3-12)	5 (2, 12)	4 (2-8)	<0.001[c]
Length of stay ratio (IQR)	0.87 (0.51-1.39)	0.92 (0.57-1.43)	0.90 (0.55-1.44)	0.82 (0.49-1.37)	0.81 (0.49-1.28)	<0.001[c]
PICU days[a]	2 (1-5)	2 (1-4)	2 (1-6)	2 (1-4)	2 (1-4)	<0.001[c]
Patients with PICU complication[b]	3,689 (7.6)	258 (6.4)	1,825 (8.3)	795 (9.0)	811 (6.0)	<0.001[d]
PICU complications	4,625	307	2,324	970	1,024	---
CLABSIs[b]	462 (1.0)	30 (0.7)	197 (0.9)	101 (1.1)	134 (1.0)	0.10[d]
VAPs[b]	200 (0.4)	5 (0.1)	66 (0.3)	60 (0.7)	69 (0.5)	<0.001[d]
Cardiac arrest[b]	381 (0.8)	29 (0.7)	182 (0.8)	81 (0.9)	89 (0.7)	0.20[d]
Renal replacement therapy[b]	877 (1.8)	65 (1.6)	407 (1.8)	277 (3.1)	128 (1.0)	<0.001[d]
Vasoactive infusion[b]	13,826 (29)	1,356 (34)	6,756 (31)	3,172 (36)	2,542 (19)	<0.001[d]
ECMO[b]	534 (1.1)	49 (1.2)	233 (1.1)	109 (1.2)	143 (1.1)	0.50[d]
Mortality[b]	1,240 (2.6%)	113 (2.8%)	583 (2.6%)	252 (2.8%)	292 (2.2%)	0.01[d]

[a] mean (IQR); [b] n (%); [c] Kruskal-Wallis Rank Sum test; [d] Pearson Chi-squared test. Abbreviations: ECMO, extracorporeal membrane oxygenation; PICU, intensive care unit.

© 2025 Taylor WM et al. *JAMA Network Open*.

eTable 6. Patient Demographics Among Agency Groups 2021

Characteristic[1]	Overall	Group 1	Group 2	Group 3	Group 4
**N**	**57,956**	**5,432**	**28,516**	**9,612**	**14,396**
Number of PICUs	19	5	9	2	3
Proportion of agency nurse staff, %	--	0	0.1-5.0	5.1-10	>10
Admit age (months)[a]	47 (10-143)	43 (9-135)	43 (9-137)	49 (11-146)	56 (13-151)
Gender[b]					
Female	25,998 (45)	2,415 (44)	12,846 (45)	4,316 (45)	6,421 (45)
Male	31,916 (55)	3,016 (56)	15,653 (55)	5,280 (55)	7,967 (55)
Unspecified	42 (<0.1)	1 (<0.1)	17 (<0.1)	16 (0.2)	8 (<0.1)
Complex chronic condition[b]	36,761 (63)	3,048 (56)	18,393 (65)	6,010 (63)	9,310 (65)
Race[b]					
Asian	2,299 (4.0)	125 (2.3)	962 (3.4)	405 (4.2)	807 (5.6)
American Indian	262 (0.5)	21 (0.4)	78 (0.3)	54 (0.6)	109 (0.8)
Black	12,288 (21)	1,067 (20)	7,593 (27)	2,490 (26)	1,138 (7.9)
Pacific Islander	277 (0.5)	20 (0.4)	65 (0.2)	12 (0.1)	180 (1.3)
White	35,474 (61)	3,966 (73)	16,270 (57)	6,101 (63)	9,137 (63)
Other Race	5,622 (9.7)	150 (2.8)	1,784 (6.3)	507 (5.3)	3,181 (22)
% nurse turnover[a]	18.5 (16.8-22.9)	14.9 (14.9-19.5)	16.9 (14.5-19.3)	17.8 (15.9-17.8)	22.9 (18.5-22.9)
Severity level[c]	2.93 (0.96)	2.84 (0.95)	2.95 (0.95)	3.10 (0.93)	2.81 (0.97)
Expected length of stay[a]	5 (3-11)	5 (3-9)	6 (3-11)	6 (3-12)	5 (3-9)

[a] median (IQR); [b] n (%); [c] mean (SD)

eTable 7. Outcomes by the Proportion of Agency Nurse Staff for 2021

Characteristic[1]	Overall	Group 1	Group 2	Group 3	Group 4	*P*
**N**	**57,956**	**5,432**	**28,516**	**9,612**	**14,396**	**---**
Number of PICUs	19	5	9	2	3	---
Length of stay[a]	4 (2-10)	4 (3-9)	5 (3-11)	5 (3-10)	4 (2-8)	<0.001[c]
Length of stay ratio (IQR)	0.86 (0.52-1.36)	0.93 (0.58-1.46)	0.89 (0.55-1.41)	0.82 (0.50-1.29)	0.78 (0.49-1.24)	<0.001[c]
PICU days[a]	2 (1-5)	2 (1-4)	2 (1-5)	2 (1-4)	2 (1-4)	<0.001[c]
Patients with PICU complication[b]	4,814 (8.3)	318 (5.9)	2,448 (8.6)	793 (8.3)	1,255 (8.7)	<0.001[d]
PICU complications	6,090	376	3,164	1,003	1,547	---
CLABSIs[b]	508 (0.9)	49 (0.9)	233 (0.8)	86 (0.9)	140 (1.0)	0.40[d]
VAPs[b]	228 (0.4)	2 (<0.1)	83 (0.3)	79 (0.8)	64 (0.4)	<0.001[d]
Cardiac arrest[b]	444 (0.8)	39 (0.7)	236 (0.8)	83 (0.9)	86 (0.6)	0.04[d]
Renal replacement therapy[b]	821 (1.4)	62 (1.1)	447 (1.6)	184 (1.9)	128 (0.9)	<0.001[d]
Vasoactive infusion[b]	14,653 (25)	1,675 (31)	7,761 (27)	2,655 (28)	2,562 (18)	<0.001[d]
ECMO[b]	654 (1.1)	63 (1.2)	313 (1.1)	122 (1.3)	156 (1.1)	0.50[d]
Mortality[b]	1,371 (2.4)	138 (2.5)	689 (2.4)	250 (2.6)	294 (2.0)	0.02[d]

[a] mean (IQR); [b] n (%); [c] Kruskal-Wallis Rank Sum test; [d] Pearson Chi-squared test. Abbreviations: ECMO, extracorporeal membrane oxygenation; PICU, intensive care unit.

© 2025 Taylor WM et al. *JAMA Network Open*.

eTable 8. Patient Demographics Among Agency Groups 2022

Characteristic[1]	Overall	Group 1	2	3	4
**N**	**51,207**	**5,417**	**20,024**	**10,049**	**15,717**
Number of PICUs	17	2	3	2	10
Proportion of agency nurse staff, %	--	0	0.1-5.0	5.1-10	>10
Admit age (months)[a]	44 (10-133)	36 (7-115)	43 (8-131)	43 (10-132)	51 (12-140)
Gender[b]					
Female	22,877 (45)	2,378 (44)	8,955 (45)	4,501 (45)	7,043 (45)
Male	28,304 (55)	3,039 (56)	11,065 (55)	5,533 (55)	8,667 (55)
Unspecified	26 (<0.1)	0 (0)	4 (<0.1)	15 (0.1)	7 (<0.1)
Complex chronic condition[b]	31,396 (61)	2,872 (53)	12,891 (64)	5,990 (60)	9,643 (61)
Race[b]					
Asian	2,250 (4.4)	134 (2.5)	694 (3.5)	511 (5.1)	911 (5.8)
American Indian	309 (0.6)	30 (0.6)	69 (0.3)	59 (0.6)	151 (1.0)
Black	9,049 (18)	1,067 (20)	4,022 (20)	2,649 (26)	1,311 (8.3)
Pacific Islander	305 (0.6)	22 (0.4)	54 (0.3)	27 (0.3)	202 (1.3)
White	32,730 (64)	3,848 (71)	12,593 (63)	6,318 (63)	9,971 (63)
Other Race	5,064 (9.9)	178 (3.3)	1,163 (5.8)	461 (4.6)	3,262 (21)
% nurse turnover[a]	17.6 (15.0-19.7)	15.0 (14.8-15.0)	17.6 (14.3-17.6)	15.3 (15.3-19.8)	18.1 (17.7-21.1)
Severity level[a]	2.95 (0.95)	2.87 (0.95)	2.97 (0.95)	3.07 (0.92)	2.86 (0.95)
Expected length of stay[a]	5 (3-10)	5 (3-9)	6 (4-11)	6 (3-11)	5 (3-9)

[a] median (IQR); [b] n (%); [c] Kruskal-Wallis Rank Sum test

eTable 9. Outcomes by the Proportion of Agency Nurse Staff for 2022

Characteristic[1]	Overall	Group 1	2	3	4	*P*
**N**	51,207	5,417	20,024	10,049	15,717	---
Number of PICUs	17	2	3	2	10	---
Length of stay[a]	4 (2-9)	4 (3-8)	5 (3-10)	4 (2-10)	4 (2-8)	<0.001[c]
Length of stay ratio (IQR)	0.84 (0.50-1.30)	0.93 (0.59-1.40)	0.86 (0.52-1.34)	0.20 (0.49-1.32)	0.78 (0.49-1.24)	<0.001[c]
PICU days[a]	5.0 (1.0-4.0)	4.1 (1.0-4.0)	5.8 (1.0-5.0)	4.6 (1.0-4.0)	4.6 (1.0-4.0)	<0.001[c]
Patients with PICU complication[b]	4,428 (8.6)	302 (5.6)	1,795 (9.0)	826 (8.2)	1,505 (9.6)	<0.001[d]
PICU complications	5,484	348	2,278	1,010	1,848	---
CLABSIs[b]	422 (0.8)	54 (1.0)	149 (0.7)	70 (0.7)	149 (0.9)	0.037[d]
VAPs[b]	188 (0.4)	7 (0.1)	67 (0.3)	41 (0.4)	73 (0.5)	0.004[d]
Cardiac arrest[b]	341 (0.7)	36 (0.7)	132 (0.7)	82 (0.8)	91 (0.6)	0.20[d]
Renal replacement therapy[b]	574 (1.1)	78 (1.4)	178 (0.9)	172 (1.7)	146 (0.9)	<0.001[d]
Vasoactive infusion[b]	11,714 (23)	1,625 (30)	5,001 (25)	2,218 (22)	2,870 (18)	<0.001[d]
ECMO[b]	507 (1.0)	45 (0.8)	193 (1.0)	108 (1.1)	161 (1.0)	0.50[d]
Mortality[b]	1,121 (2.2)	121 (2.2)	466 (2.3)	260 (2.6)	274 (1.7)	<0.001[d]

[a] mean (IQR); [b] n (%); [c] Kruskal-Wallis Rank Sum test; [d] Pearson Chi-squared test. Abbreviations: ECMO, extracorporeal membrane oxygenation; PICU, intensive care unit.

© 2025 Taylor WM et al. *JAMA Network Open*.

eTable 10. Demographics Among Groups Defined by Nurse Turnover

Characteristic	Overall	Group<br>1<br>(<12.5%)	2<br>(12.5-15.0%)	3<br>(15.0-17.5%)	4<br>(>17.5%)
**N**	203,161	18,294	73,822	64,874	46,171
Number of PICUs	20	3	7	5	5
Proportion of nurse turnover, %	--	<12.5	12.5-15.0	15.0-17.5	>17.5
Admit age (months)[a]	46 (9 -139)	47 (11-140)	42 (7-135)	48 (10-139)	48 (11-143)
Gender[b]					
Female	91,668 (45)	8,326 (46)	33,349 (45)	29,084 (45)	20,909 (45)
Male	111,366 (55)	9,956 (54)	40,428 (55)	35,745 (55)	25,237 (55)
Unspecified	127 (<0.1)	12 (<0.1)	45 (<0.1)	45 (<0.1)	25 (<0.1)
Complex chronic condition[b]	129,735 (64)	12,272 (67)	49,281 (67)	39,245 (60)	28,937 (63)
Race[b]					
Asian	8,736 (4.3)	1,089 (6.0)	3,055 (4.1)	3,386 (5.2)	1,206 (2.6)
American Indian	963 (0.5)	24 (0.1)	318 (0.4)	296 (0.5)	325 (0.7)
Black	40,205 (20)	3,504 (19)	14,246 (19)	8,738 (13)	13,717 (30)
Pacific Islander	1,247 (0.6)	177 (1.0)	448 (0.6)	437 (0.7)	185 (0.4)
White	125,734 (62)	8,996 (49)	48,782 (66)	42,722 (66)	25,234 (55)
Other Race	21,038 (10)	3,955 (22)	3,555 (4.8)	8,617 (13)	4,911 (11)
Agency nurse proportion[a]	0.03 (0.00-0.10)	0.02 (0.01-0.06)	0.01 (0.00-0.14)	0.06 (0.01-0.13)	0.07 (0.02-0.08)
Severity level[a]	2.91 (0.97)	2.89 (0.98)	2.97 (0.97)	2.82 (0.99)	2.96 (0.92)
Expected length of stay, days[a]	5 (3-11)	5 (3-10)	6 (3-12)	5 (3-9)	5 (3-9)

[a] median (IQR); [b] n (%); [c] mean (sd)

eTable 11. Outcomes Defined by Nurse Turnover

	Overall	Group<br>1<br>(<12.5%)	2<br>(12.5-15.0%)	3<br>(15.0-17.5%)	4<br>(>17.5%)	*P*
**N**	203,161	18,294	73,822	64,874	46,171	
Number of PICUs	20	3	7	5	5	
Length of stay, days[a]	4 (2-10)	5 (3-10)	5 (3-12)	4 (2, 8)	4 (2, 9)	<0.001[c]
Length of stay ratio (IQR)	0.87 (0.52-1.36)	0.90 (0.57-1.39)	0.89 (0.53- 1.42)	0.83 (0.50-1.29)	0.87 (0.52-1.34)	<0.001[c]
PICU days[a]	2 (1-5)	2 (1-5)	2 (1-5)	2 (1-4)	2 (1-5)	<0.001[c]
Patients with PICU complication[b]	15,613 (7.6)	1,552 (8.4)	5,936 (8.0)	4,079 (6.3)	4,046 (8.8)	<0.001[d]
PICU complications	19,496	2,201	7,528	4,978	4,789	---
CLABSIs[b]	1,688 (0.8)	136 (0.7)	804 (1.1)	432 (0.7)	316 (0.7)	<0.001[d]
VAPs[b]	760 (0.4)	58 (0.3)	291 (0.4)	276 (0.4)	135 (0.3)	0.002[d]
Cardiac arrest[b]	1,466 (0.7)	134 (0.7)	633 (0.9)	367 (0.6)	332 (0.7)	0.004[d]
Renal replacement therapy[b]	2,975 (1.5)	312 (1.7)	1,289 (1.7)	713 (1.1)	661 (1.4)	<0.001[d]
Vasoactive infusion[b]	48,848 (24)	5,387 (29)	23,362 (32)	9,829 (15)	10,270 (22)	<0.001[d]
ECMO[b]	2,073 (1.0)	199 (1.1)	826 (1.1)	519 (0.8)	529 (1.1)	<0.001[d]
Mortality[b]	4,810 (2.4)	377 (2.1)	1,871 (2.5)	1,429 (2.2)	1,133 (2.5)	<0.001[d]

[a] median (IQR); [b] n (%); [c] Kruskal-Wallis Rank Sum test; [d] Pearson Chi-squared test. Abbreviations: ECMO, extracorporeal membrane oxygenation; PICU, intensive care unit.

© 2025 Taylor WM et al. *JAMA Network Open*.

eTable 12. Patient Demographics Among Nursing Groups 2019

Characteristic	Overall	Group 1 (<12.5%)	Group 2 (12.5-15.0%)	Group 3 (15.0-17.5%)	Group 4 (>17.5%)
N	50,557	6,621	10,292	17,666	15,978
Number of PICUs	16	3	4	4	5
Proportion of nurse turnover, %	--	<12.5	12.5-15.0	15.0-17.5	>17.5
Admit age (months)[a]	39 (8-128)	42 (11-129)	31 (5-124)	43 (9-130)	38 (8-128)
Gender[b]					
Female	22,912 (45)	2,985 (45)	4,681 (45)	8,040 (46)	7,206 (45)
Male	27,617 (55)	3,632 (55)	5,610 (55)	9,613 (54)	8,762 (55)
Unspecified	28 (<0.1)	4 (<0.1)	1 (<0.1)	13 (<0.1)	10 (<0.1)
Complex chronic condition[b]	31,884 (63)	4,398 (66)	7,095 (69)	10,323 (58)	10,068 (63)
Race[b]					
Asian	2,335 (4.6)	377 (5.7)	412 (4.0)	1,110 (6.3)	436 (2.7)
American Indian	195 (0.4)	15 (0.2)	30 (0.3)	53 (0.3)	97 (0.6)
Black	10,170 (20)	1,093 (17)	1,966 (19)	2,584 (15)	4,527 (28)
Pacific Islander	399 (0.8)	88 (1.3)	101 (1.0)	76 (0.4)	134 (0.8)
White	30,915 (61)	3,018 (46)	6,857 (67)	11,627 (66)	9,413 (59)
Other Race	5,274 (10)	1,901 (29)	640 (6.2)	1,884 (11)	849 (5.3)
Agency nurse proportion[a]	0.02 (0.00-0.08)	0.01 (0.01-0.06)	0.00 (0.00-0.00)	0.00 (0.00-0.13)	0.07 (0.0-0.08)
Severity level[a]	2.86 (0.99)	2.83 (0.99)	2.93 (0.97)	2.69 (1.03)	3.01 (0.92)
Expected length of stay, days[a]	5 (3-11)	5 (3-9)	7 (4-12)	4 (3-9)	5 (3-12)

[a] median (IQR); [b] n (%); [c] mean (sd)

eTable 13. Patient Outcomes Among Nursing Groups 2019

	Overall	Group 1 (<12.5%)	Group 2 (12.5-15.0%)	Group 3 (15.0-17.5%)	Group 4 (>17.5%)	*P*
N	50,557	6,621	10,292	17,666	50,557	
Number of PICUs	16	3	4	4	16	
Length of stay, days[a]	4 (2-10)	4 (-9)	6, (3, 14)	4 (2-8)	5, (3, 10)	<0.001[c]
Length of stay ratio (IQR)	0.89 (0.55-1.41)	0.91 (0.57-1.37)	0.97 (0.60-1.55)	0.88 (0.52-1.38)	0.86 (0.53-1.35)	<0.001[c]
PICU days[a]	2 (1-5)	2, (1, 4)	3 (1-6)	2 (1-4)	2 (-5)	<0.001[c]
Patients with PICU complication[b]	3,111 (6.1)	492 (7.4)	691 (6.7)	851 (4.8)	1,079 (6.7)	<0.001[d]
PICU complications	3815	708	878	983	1267	---
CLABSIs[b]	347 (0.7)	45 (0.7)	106 (1.0)	117 (0.7)	79 (0.5)	<0.001[d]
VAPs[b]	150 (0.3)	19 (0.3)	23 (0.2)	31 (0.2)	77 (0.5)	<0.001[d]
Cardiac arrest[b]	338 (0.7)	31 (0.5)	90 (0.9)	79 (0.4)	138 (0.9)	<0.001[d]
Renal replacement therapy[b]	763 (1.5)	101 (1.5)	162 (1.6)	224 (1.3)	276 (1.7)	0.006[d]
Vasoactive infusion[b]	11,187 (22)	1,824 (28)	2,828 (27)	2,942 (17)	3,593 (22)	<0.001[d]
ECMO[b]	453 (0.9)	59 (0.9)	100 (1.0)	89 (0.5)	205 (1.3)	<0.001[d]
Mortality[b]	1,202 (2.4)	138 (2.1)	272 (2.6)	346 (2.0)	446 (2.8)	<0.001[d]

© 2025 Taylor WM et al. *JAMA Network Open*.

eTable 14. Patient Demographics Among Nursing Groups 2020

Characteristic	Overall	Group<br>1<br>(<12.5%)	2<br>(12.5-15.0%)	3<br>(15.0-17.5%)	4<br>(>17.5%)
N	43,441	7,393	16,408	10,611	9,029
Number of PICUs	18	4	5	5	4
Proportion of nurse turnover, %	--	<12.5	12.5-15.0	15.0-17.5	>17.5
Admit age (months)[a]	54 (10-149)	54 (10-150)	54 (10-146)	50 (9-148)	59 (11-153)
Gender[b]					
Female	19,881 (46)	3,422 (46)	7,440 (45)	4,865 (46)	4,154 (46)
Male	23,529 (54)	3,970 (54)	8,946 (55)	5,741 (54)	4,872 (54)
Unspecified	31 (<0.1)	1 (<0.1)	22 (0.1)	5 (<0.1)	3 (<0.1)
Complex chronic condition[b]	29,694 (68)	5,333 (72)	10,904 (6%)	7,226 (68)	6,231 (69)
Race[b]					
Asian	1,852 (4.3)	332 (4.5)	896 (5.5)	361 (3.4)	263 (2.9)
American Indian	197 (0.5)	6 (<0.1)	61 (0.4)	106 (1.0)	24 (0.3)
Black	8,698 (20)	1,323 (18)	2,683 (16)	1,270 (12)	3,422 (38)
Pacific Islander	266 (0.6)	68 (0.9)	35 (0.2)	148 (1.4)	15 (0.2)
White	26,615 (61)	4,098 (55)	10,753 (6%)	7,236 (68)	4,528 (50)
Other Race	5,078 (12)	1,436 (19)	1,533 (9.3)	1,697 (16)	412 (4.6)
Agency nurse proportion[a]	0.02 (0.00-0.06)	0.01 (0.00-0.04)	0.02 (0.01-0.11)	0.06 (0.01-0.08)	0.00 (0.00-0.00)
Severity level[c]	2.93 (0.97)	2.95 (0.97)	2.83 (1.01)	3.04 (0.91)	2.95 (0.96)
Expected length of stay, days[a]	6 (3-12)	6 (3-12)	5 (3-11)	6 (3-12)	6 (3-12)

[a] median (IQR); [b] n (%); [c] mean (sd)

eTable 15. Patient Outcomes Among Nursing Groups 2020

	Overall	Group<br>1<br>(<12.5%)	2<br>(12.5-15.0%)	3<br>(15.0-17.5%)	4<br>(>17.5%)	*P*
N	43,441	7,393	16,408	10,611	9,029	
Number of PICUs	18	4	5	5	4	
Length of stay, days[a]	5 (2-11)	5 (3-11)	4 (2-10)	5 (3-11)	4 (2-10)	<0.001[c]
Length of stay ratio (IQR)	0.87 (0.51-1.38)	0.88 (0.56-1.37)	0.85 (0.49 1.39)	0.89 (0.55-1.38)	0.86 (0.49-1.38)	<0.001[c]
PICU days[a]	2 (1-5)	3 (1-5)	2 (1-5)	2 (1-4)	2 (1-5)	<0.001[c]
Patients with PICU complication[b]	3,258 (7.5)	605 (7.2)	1,31 (6.9)	823 (7.8)	699 (7.7)	<0.001[d]
PICU complications	4,086	812	1,388	1,041	845	---
CLABSIs[b]	411 (0.9)	71 (1.0)	171 (1.0)	99 (0.9)	70 (0.8)	0.20[d]
VAPs[b]	194 (0.4)	31 (0.4)	75 (0.5)	65 (0.6)	23 (0.3)	0.003[d]
Cardiac arrest[b]	343 (0.8)	64 (0.9)	114 (0.7)	103 (1.0)	62 (0.7)	0.044[d]
Renal replacement therapy[b]	817 (1.9)	143 (1.9)	355 (2.2)	148 (1.4)	171 (1.9)	<0.001[d]
Vasoactive infusion[b]	11,294 (26)	2,312 (31)	4,053 (25)	2,664 (25)	2,265 (25)	<0.001[d]
ECMO[b]	459 (1.1)	82 (1.1)	135 (0.8)	141 (1.3)	101 (1.1)	<0.001[d]
Mortality[b]	1,116 (2.6)	153 (2.1)	376 (2.3)	323 (3.0)	264 (2.9)	<0.001[d]

© 2025 Taylor WM et al. *JAMA Network Open*.

eTable 16. Patient Demographics Among Nursing Groups 2021

Characteristic	Overall	Group<br>1<br>(<12.5%)	2<br>(12.5-15.0%)	3<br>(15.0-17.5%)	4<br>(>17.5%)
N	57,956	14,151	14,580	10,656	18,569
Number of PICUs	19	2	7	5	5
Proportion of nurse turnover, %	--	<12.5	12.5-15.0	15.0-17.5	>17.5
Admit age (months)[a]	47 (10-143)	46 (10-139)	38 (6-140)	47 (10-141)	55 (13-149)
Gender[b]					
Female	25,998 (45)	6,325 (45)	6,570 (45)	4,798 (45)	8,305 (45)
Male	31,916 (55)	7,810 (55)	8,001 (55)	5,856 (55)	10,249 (55)
Unspecified	42 (<0.1)	16 (0.1)	9 (<0.1)	2 (<0.1)	15 (<0.1)
Complex chronic condition[b]	36,761 (63)	9,588 (68)	9,139 (63)	7,062 (66)	10,972 (59)
Race[b]					
Asian	2,299 (4.0)	582 (4.1)	500 (3.4)	379 (3.6)	838 (4.5)
American Indian	262 (0.5)	60 (0.4)	74 (0.5)	37 (0.3)	91 (0.5)
Black	12,288 (21)	2,786 (20)	2,919 (20)	2,178 (20)	4,405 (24)
Pacific Islander	277 (0.5)	24 (0.2)	95 (0.7)	121 (1.1)	37 (0.2)
White	35,474 (61)	8,119 (57)	10,180 (70)	6,981 (66)	10,194 (55)
Other Race	5,622 (9.7)	1,771 (13)	150 (1.0)	999 (9.4)	2,702 (15)
Agency nurse proportion[a]	0.02 (0.00-0.04)	0.00 (0.00-0.02)	0.01 (0.01-0.04)	0.00 (0.00-0.02)	0.06 (0.03- 0.07)
Severity level[c]	2.93 (0.96)	2.99 (0.96)	2.97 (0.97)	3.02 (0.91)	2.79 (0.96)
Expected length of stay, days[a]	5 (3-11)	6 (3-12)	6 (3-12)	6 (3-12)	4 (3-9)

[a] median (IQR); [b] n (%); [c] mean (sd)

eTable 17. Patient Outcomes Among Nursing Groups 2021

	Overall	Group<br>1<br>(<12.5%)	2<br>(12.5-15.0%)	3<br>(15.0-17.5%)	4<br>(>17.5%)	*P*
N	57,956	14,151	14,580	10,656	18,569	
Number of PICUs	19	2	7	5	5	
Length of stay, days[a]	4 (2-10)	5 (3-11)	5(2-11)	5 (3-12)	4 (2-7)	<0.001[c]
Length of stay ratio (IQR)	0.86 (0.52-1.36)	0.88 (0.56-1.38)	0.81 (0.49- 1.29)	0.93 (0.59-1.52)	0.81 (0.49-1.28)	<0.001[c]
PICU days[a]	2 (1-5)	2 (1-5)	2 (1-5)	2 (1-4)	2 (1-4)	<0.001[c]
Patients with PICU complication[b]	4,814 (8.3)	1,317(9.3)	1,188 (8.1)	845 (7.9)	1,464 (7.8)	<0.001[d]
PICU complications	6,090	1,729	1,546	1,099	1,716	---
CLABSIs[b]	508 (0.9)	130 (0.9)	157 (1.1)	90 (0.8)	131 (0.7)	0.004[d]
VAPs[b]	228 (0.4)	65 (0.5)	104 (0.7)	33 (0.3)	26 (0.1)	<0.001[d]
Cardiac arrest[b]	444 (0.8)	140 (1.0)	125 (0.9)	85 (0.8)	94 (0.5)	<0.001[d]
Renal replacement therapy[b]	821 (1.4)	224 (1.6)	213 (1.5)	182 (1.7)	202 (1.1)	<0.001[d]
Vasoactive infusion[b]	14,653 (25)	5,164 (36)	3,882 (27)	2,374 (22)	3,233 (17)	<0.001[d]
ECMO[b]	654 (1.1)	223 (1.6)	152 (1.0)	118 (1.1)	161 (0.9)	<0.001[d]
Mortality[b]	1,371 (2.4)	324 (2.3)	376 (2.6)	331 (3.1)	340 (1.8)	<0.001[d]

© 2025 Taylor WM et al. *JAMA Network Open*.

eTable 18. Patient Demographics Among Nursing Groups 2022

Characteristic	Overall	Group<br>1<br>(<12.5%)	2<br>(12.5-15.0%)	3<br>(15.0-17.5%)	4<br>(>17.5%)
N	51,207	7,831	12,818	15,085	15,473
Number of PICUs	18	3	4	4	6
Proportion of nurse turnover, %	--	<12.5	12.5-15.0	15.0-17.5	>17.5
Admit age (months)[a]	44 (10-133)	37 (8-119)	38 (7-128)	57 (12-145)	41 (11-129)
Gender[b]					
Female	22,877 (45)	3,462 (44)	5,759 (45)	6,745 (45)	6,911 (45)
Male	28,304 (55)	4,368 (56)	7,050 (55)	8,335 (55)	8,551 (55)
Unspecified	26 (<0.1)	1 (<0.1)	9 (<0.1)	5 (<0.1)	11 (<0.1)
Complex chronic condition[b]	31,396 (61)	5,036 (64)	7,687 (60)	9,248 (61)	9,425 (61)
Race[b]					
Asian	2,250 (4.4)	376 (4.8)	489 (3.8)	892 (5.9)	493 (3.2)
American Indian	309 (0.6)	18 (0.2)	71 (0.6)	94 (0.6)	126 (0.8)
Black	9,049 (18)	1,321 (17)	2,819 (22)	1,594 (11)	3,315 (21)
Pacific Islander	305 (0.6)	25 (0.3)	41 (0.3)	161 (1.1)	78 (0.5)
White	32,730 (64)	4,694 (60)	8,614 (67)	9,968 (66)	9,454 (61)
Other Race	5,064 (9.9)	637 (8.1)	56 (0.4)	2,010 (13)	2,361 (15)
Agency nurse proportion[a]	0.15 (0.01-0.23)	0.08 (0.00- 0.10)	0.04 (0.00-0.25)	0.20 (0.01-0.22)	0.15 (0.11-0.23)
Severity level[c]	2.95 (0.95)	2.96 (0.93)	3.02 (0.95)	2.74 (0.99)	3.08 (0.88)
Expected length of stay, days[a]	5 (3-10)	6 (3-10)	6 (3-12)	5 (3-9)	5 (3-9)

[a] median (IQR); [b] n (%); [c] mean (sd)

eTable 19. Patient Outcomes Among Nursing Groups 2022

	Overall	Group<br>1<br>(<12.5%)	2<br>(12.5-15.0%)	3<br>(15.0-17.5%)	4<br>(>17.5%)	*P*
N	51,207	7,831	12,818	15,085	15,473	
Number of PICUs	18	3	4	4	6	
Length of stay, days[a]	4 (2-9)	5 (3-11)	5 (2-10)	4 (2-8)	4 (3-9)	<0.001[c]
Length of stay ratio (IQR)	0.84 (0.50-1.30)	0.93 (0.58-1.43)	0.82 (0.49-1.35)	0.78 (0.49-1.22)	0.84 (0.52-1.28)	<0.001[c]
PICU days[a]	2.0 (1.0-4.0)	2.0 (1.0-5.0)	2.0 (1.0-5.0)	2.0 (1.0-4.0)	2.0 (1.0-4.0)	<0.001[c]
Patients with PICU complication[b]	4,428 (8.6)	585 (7.6)	1,059 (8.3)	1,126 (7.5)	1,648 (10.7)	<0.001[d]
PICU complications	5,484	786	1,289	1,458	1,951	---
CLABSIs[b]	422 (0.8)	49 (0.6)	116 (0.9)	165 (1.1)	92 (0.6)	<0.001[d]
VAPs[b]	188 (0.4)	10 (0.1)	37 (0.3)	73 (0.5)	68 (0.4)	<0.001[d]
Cardiac arrest[b]	341 (0.7%)	61 (0.8%)	91 (0.7%)	100 (0.7%)	89 (0.6%)	0.30[d]
Renal replacement therapy[b]	574 (1.1%)	98 (1.3%)	176 (1.4%)	158 (1.0%)	142 (0.9%)	0.002[d]
Vasoactive infusion[b]	11,714 (23%)	2,884 (37%)	3,523 (27%)	2,969 (20%)	2,338 (15%)	<0.001[d]
ECMO[b]	507 (1.0%)	82 (1.0%)	130 (1.0%)	134 (0.9%)	161 (1.0%)	0.50[d]
Mortality[b]	1,121 (2.2%)	188 (2.4%)	322 (2.5%)	265 (1.8%)	346 (2.2%	<0.001[d]

© 2025 Taylor WM et al. *JAMA Network Open*.

eTable 20. Demographics Among Units With Stable or Variable Agency Staffing Between 2019 and 2022

Characteristic[1]	Overall	Stable Agency	Agency Variability
**N**	**218,789**	**98,772**	**120,017**
Number of PICUs	20	10	10
Admit age (months)[a]	45 (9-138)	43 (8-136)	47 (11-139)
Gender[b]			
Female	98,664 (45)	44,684 (45)	53,980 (45)
Male	119,997 (55)	54,048 (55%)	65,949 (55%)
Unspecified	128 (<0.1)	40 (<0.1%)	88 (<0.1%)
Complex chronic condition[b]	140,500 (64)	62,121 (63%)	78,379 (65%)
Race[b]			
Asian	9,170 (4.2)	3,273 (3.3%)	5,897 (4.9%)
American Indian	1,092 (0.5)	329 (0.3%)	763 (0.6%)
Black	42,313 (19)	24,840 (25%)	17,473 (15%)
Pacific Islander	1,268 (0.6)	398 (0.4%)	870 (0.7%)
White	134,811 (62)	60,005 (61%)	74,806 (62%)
Other Race	22,952 (10)	5,838 (5.9%)	17,114 (14%)
% nurse turnover[a]	15.0 (12.4-18.1)	14.9 (11.1-17.6)	15.9 (13.0-19.0)
2019	13.0 (10.5-14.9)	11.1 (10.4-14.9)	13.4 (12.1-14.7)
2020	12.4 (11.4-14.3)	11.5 (9.7-16.2)	12.4 (11.8-13.2)
2021	18.5 (16.8-22.9)	16.9 (14.9-19.5)	18.5 (17.6-22.9)
2022	17.6 (15.0-19.6)	15.0 (14.8-17.6)	18.1 (15.3-19.8)
Severity level[c]	2.91 (0.97)	2.90 (0.96)	2.92 (0.97)
Expected length of stay[a]	5 (3-11)	5 (3-11)	5 (3-11)

[a] median (IQR); [b] n (%); [c] mean (SD)

eTable 21. Outcomes Among Units With Stable or Variable Agency Staffing Between 2019 and 2022

Characteristic[1]	Overall	Stable Staffing	Variable Staffing	*P*
**N**	218,789	98,772	120,017	---
Number of PICUs	20	10	10	---
Length of stay[a]	4 (2-10)	5 (3-10)	4 (2-10)	<0.001[c]
Length of stay ratio (IQR)	0.87 (0.52-1.37)	0.90 (0.56-1.41)	0.84 (0.50-1.33)	<0.001[c]
PICU days[a]	2 (1-5)	2 (1-5)	2 (1-4)	<0.001[c]
Patients with an PICU complication[b]	16,836 (7.7)	7,141 (7.2)	9,695 (8.1)	<0.001[d]
PICU complications	21,033	9,001	12,032	---
CLABSIs[b]	1,839 (0.8%)	801 (0.8%)	1,038 (0.9%)	0.20[d]
VAPs[b]	796 (0.4%)	248 (0.3%)	548 (0.5%)	<0.001[d]
Cardiac arrest [b]	1,561 (0.7)	757 (0.8)	804 (0.7)	0.008[d]
Renal replacement therapy [b]	3,143 (1.4)	1,587 (1.6)	1,556 (1.3)	<0.001[d]
Vasoactive infusion [b]	55,481 (25)	26,603 (27)	28,878 (24)	<0.001[d]
ECMO [b]	2,279 (1.0)	955 (1.0)	1,324 (1.1)	0.002[d]
Mortality [b]	5,131 (2.3)	2,413 (2.4)	2,718 (2.3)	0.006[d]

[a] mean (IQR); [b] n (%); [c] Kruskal-Wallis Rank Sum test; [d] Pearson Chi-squared test. Abbreviations: ECMO, extracorporeal membrane oxygenation; PICU, intensive care unit.

© 2025 Taylor WM et al. *JAMA Network Open*.

eFigure. Alluvial Diagrams for Hospital Proportion Agency Staff and Nurse Turnover

Each vertical bar represents a study year. The alluvial bands represent the percentage change of agency staffing through the study years. Stable Agency represents a <10% increase in an PICU's percent agency staffing over the study years. Agency Variability represents a ≥10% increase in an PICU's agency staffing over the study years. Each PICU is followed as one band to depict stability and change across the study years. A) Groups 1-4 represent the percentage of agency staffing among the PICU during that study year, with Group 1 = 0.0%, Group 2 0.1-5.0%, Group 3 5.1-10.0% and Group 4 >10.0% agency staffing, respectively. B) Groups 1-4 RN turnover percentages varied per study year and represent clustered groups per year based on nearest neighbor. The minimum turnover percentage through the study years was 5.2% in 2019 to a maximum of 30.9% in 2021.

A) Agency Staffing Percentage

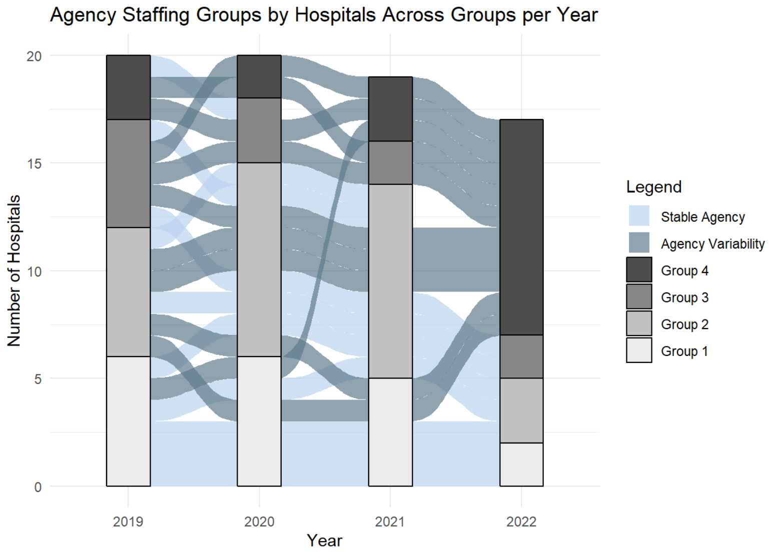

B) RN Turnover Rate

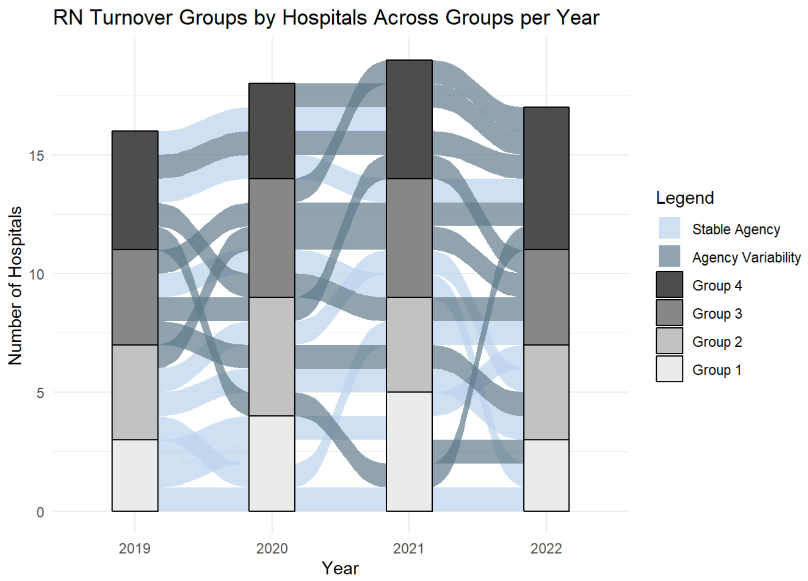

© 2025 Taylor WM et al. *JAMA Network Open*.
