# Supplementary material for: Pediatric Intensive Care Nurse Staffing Measures and Patient Outcomes During the COVID-19 Pandemic
Source: JAMA Netw Open. 2025 Jun 12;8(6):e2515376. doi: 10.1001/jamanetworkopen.2025.15376 (PMC12163677; doi:10.1001/jamanetworkopen.2025.15376)
Supplement: Supplement 2. — Data Sharing Statement [file jamanetwopen-e2515376-s002.pdf]

Data Sharing Statement

Taylor. Pediatric Intensive Care Nurse Staffing Measures and Patient Outcomes During the COVID-19 Pandemic. *JAMA Netw Open*. Published June 12, 2025. doi:10.1001/jamanetworkopen.2025.15376

Data
Data available: No

Additional Information
Explanation for why data not available: Due to a Data Use Agreement with Children's Hospital Association, we cannot share our data outside of member institutions.
